# *TBC1D24* genotype–phenotype correlation: Epilepsies and other neurologic features

**DOI:** 10.1212/WNL.0000000000002807

**Published:** 2016-07-05

**Authors:** Simona Balestrini, Mathieu Milh, Claudia Castiglioni, Kevin Lüthy, Mattea J. Finelli, Patrik Verstreken, Aaron Cardon, Barbara Gnidovec Stražišar, J. Lloyd Holder, Gaetan Lesca, Maria M. Mancardi, Anne L. Poulat, Gabriela M. Repetto, Siddharth Banka, Leonilda Bilo, Laura E. Birkeland, Friedrich Bosch, Knut Brockmann, J. Helen Cross, Diane Doummar, Temis M. Félix, Fabienne Giuliano, Mutsuki Hori, Irina Hüning, Hulia Kayserili, Usha Kini, Melissa M. Lees, Girish Meenakshi, Leena Mewasingh, Alistair T. Pagnamenta, Silvio Peluso, Antje Mey, Gregory M. Rice, Jill A. Rosenfeld, Jenny C. Taylor, Matthew M. Troester, Christine M. Stanley, Dorothee Ville, Magdalena Walkiewicz, Antonio Falace, Anna Fassio, Johannes R. Lemke, Saskia Biskup, Jessica Tardif, Norbert F. Ajeawung, Aslihan Tolun, Mark Corbett, Jozef Gecz, Zaid Afawi, Katherine B. Howell, Karen L. Oliver, Samuel F. Berkovic, Ingrid E. Scheffer, Fabrizio A. de Falco, Peter L. Oliver, Pasquale Striano, Federico Zara, Phillipe M. Campeau, S.M. Sisodiya

## Abstract

**Objective:**

To evaluate the phenotypic spectrum associated with mutations in *TBC1D24*.

**Methods:**

We acquired new clinical, EEG, and neuroimaging data of 11 previously unreported and 37 published patients. *TBC1D24* mutations, identified through various sequencing methods, can be found online (http://lovd.nl/TBC1D24).

**Results:**

Forty-eight patients were included (28 men, 20 women, average age 21 years) from 30 independent families. Eighteen patients (38%) had myoclonic epilepsies. The other patients carried diagnoses of focal (25%), multifocal (2%), generalized (4%), and unclassified epilepsy (6%), and early-onset epileptic encephalopathy (25%). Most patients had drug-resistant epilepsy. We detail EEG, neuroimaging, developmental, and cognitive features, treatment responsiveness, and physical examination. In silico evaluation revealed 7 different highly conserved motifs, with the most common pathogenic mutation located in the first. Neuronal outgrowth assays showed that some *TBC1D24* mutations, associated with the most severe *TBC1D24*-associated disorders, are not necessarily the most disruptive to this gene function.

**Conclusions:**

*TBC1D24*-related epilepsy syndromes show marked phenotypic pleiotropy, with multisystem involvement and severity spectrum ranging from isolated deafness (not studied here), benign myoclonic epilepsy restricted to childhood with complete seizure control and normal intellect, to early-onset epileptic encephalopathy with severe developmental delay and early death. There is no distinct correlation with mutation type or location yet, but patterns are emerging. Given the phenotypic breadth observed, *TBC1D24* mutation screening is indicated in a wide variety of epilepsies. A *TBC1D24* consortium was formed to develop further research on this gene and its associated phenotypes.

The gene *TBC1D24* is involved in regulation of synaptic vesicle trafficking and in brain and somatic development.^1–5^ It has recently been implicated in various human diseases, many of which feature epileptic seizures^1,3,6–14^; mutations can also cause nonsyndromic deafness.^15–18^

*TBC1D24* encodes a protein containing a Tre2/Bub2/Cdc16 (TBC) domain, shared by Rab GTPase-activating proteins (Rab-GAPs). TBC domain-containing proteins regulate numerous vesicular membrane-trafficking and sorting processes by modulating the activity of Rab-GTPases.^19^ TBC1D24 interacts with the ADP ribosylation factor 6 (ARF6), a small GTP-binding protein involved in membrane exchange between plasma membrane and endocytic compartments.^1,11^ The protein also contains a TLDc domain, putatively involved in oxidative stress resistance.^20^

In *TBC1D24*-associated disorders, including deafness, onychodystrophy, osteodystrophy, mental retardation, and seizures (DOORS) syndrome, a wide spectrum of epilepsies have been reported. We evaluated the types of epilepsy seen with a wide mutational spectrum in *TBC1D24*, using new data from 11 previously unreported and 37 published patients.

## METHODS

### Standard protocol approvals, registrations, and patient consents.

This study was approved by the relevant institutional ethics committees or review boards. Parental (or legal guardian) written informed consent was obtained for all affected children and adults with intellectual disability, or existing published data were collated. Authorization has been obtained for disclosure of videos (videos 1–5 on the *Neurology*® Web site at Neurology.org).

### Participants.

We collected data from a cohort of new patients and contacted physicians to seek additional information about published patients by using a standardized questionnaire. Available neuroimaging and EEG recordings were evaluated. Individuals were included if they had a confirmed *TBC1D24* mutation and had epileptic seizures within their phenotype. We report 2 additional patients (31, 32) where a clear association of phenotype with changes in *TBC1D24* could not be established: therefore, they were not included in the final analyses. In patient 31, 2 single nucleotide polymorphisms in the maternal allele were found through a next-generation sequencing panel; in patient 32, array comparative genomic hybridization showed a 16p13.3 duplication including *TBC1D24*.

### Procedures.

*TBC1D24* variants were identified through various methods, detailed in supplemental data S1. Conserved motifs were detected in MEME suite^21^ through discriminative motif discovery. Bioinformatics and in vitro modeling methods are described in supplemental data S1.

## RESULTS

### Family history.

The cohort includes 48 patients (28 men, 20 women) from 30 independent families. Twenty-seven individuals are from 9 families (figure e-1), comprising 5 sibling pairs with nonconsanguineous parents,^3,11,14,22^ 1 pair with consanguineous parents,^8^ 4 members of a large Arab-Israeli family with multiple consanguineous unions,^6^ 8 members of a large Italian family,^1,7^ and 3 members of a large Turkish family (all patients born to consanguineous parents).^9,10^ Six other sporadic patients, including one previously described,^23^ have consanguineous parents; 15 patients (31%) are isolated, from nonconsanguineous parents.

### Longevity.

Nine individuals (19%) were deceased (average age at death 37 months, range 6–96 months).^3,9,11,14^ One death (6b), at age 18 months, was defined as probable sudden unexpected death in epilepsy.^11^ The other reported causes of death were infectious episode (7a, 7c, 17a, 17b), respiratory failure (6a), status epilepticus associated with a pulmonary infection (7b), and unknown (26, 28). The average age of the living patients was 21 years in January 2015.

### Seizures and treatment responsiveness.

The types of seizures and epilepsies were diverse. Seizure types included infantile spasms and febrile convulsive, myoclonic, clonic, tonic, absence, tonic-clonic with or without apparent focal onset, and focal seizures with retained or impaired awareness. Myoclonic or clonic seizures were the most frequent seizure types (29/48, 60%), often unresponsive to medication. Myoclonic seizures were segmental (often involving eyelids, perioral region, or other facial parts) or generalized, with initially no loss of consciousness, but sometimes evolving into tonic-clonic seizures. Various other features of myoclonic seizures were described; they could be unilateral or bilateral, migrating, alternating, rhythmic, or pseudorhythmic, occurring both at rest and on maintaining posture. They often occurred in clusters, which could be very prolonged, lasting several days. In some patients, they were triggered by fatigue, drowsiness, intense and persistent stimulation (acoustic stimuli or variations in light intensity), repetitive movements, feeding, febrile episodes, constipation, or delayed medication. Eighteen patients had myoclonic epilepsy (including infantile myoclonic and progressive myoclonic epilepsies). Semiologic features of 5 patients, 4 with myoclonic epilepsy (4, 23a, 23b, 24) and 1 with familial epilepsy of infancy with migrating focal seizures (EIMFS) (6b), are shown in videos 1–5. The other patients had focal, multifocal, generalized, or unclassified epilepsy, or early-onset epileptic encephalopathy (including EIMFS). There was no marked variability of epilepsy phenotype in affected siblings.

The average age at seizure onset was 7 months (range from within 1 hour after birth to 8 years; SD 15 months). Thirty-eight (79%) individuals had had status epilepticus, either convulsive or nonconvulsive, or prolonged seizures (>5 minutes). In 19 patients, seizures or status episodes were precipitated by fever or infections.

In 30 patients, epilepsy was drug-resistant^24^; 18 patients responded well to treatment. The Italian family with familial infantile myoclonic epilepsy was drug-responsive, with 5 members (1a–1e) free from tonic-clonic seizures and with rare myoclonic seizures, on valproate or phenobarbital, while the remaining affected individuals (1f–1h) were not on any antiepileptic medication, and experienced mild myoclonic seizures triggered by repetitive movements or fatigue and rare tonic-clonic seizures (every 2–3 years). One patient (25) with generalized epilepsy was seizure-free on phenytoin and clobazam. Two siblings (12a and 12b) with focal seizures had significant improvement of seizure control after introduction of zonisamide; 1 patient (11) with infantile myoclonic epilepsy had a good response to topiramate. Patient 15 had prolonged monthly tonic-clonic seizures mostly during febrile episodes; there was dramatic improvement over the last few years, with freedom from tonic-clonic seizures for >12 months, on a combination of oxcarbazepine and sulthiame, possibly due to these drugs or less frequent febrile episodes. Another 5 patients (5c, 5d, 20a, 20b, 22) had adequate seizure control on 2 or 3 antiepileptic drugs. Details of epilepsy phenotypes are presented in table e-1.

### EEG and neuroimaging.

Results are reported in table e-2. Thirteen patients had a normal interictal EEG record. Various features, including slow background activity and multifocal paroxysmal abnormalities, were described in 35 patients. Only 2 patients had a photoparoxysmal EEG response (1b, 1h). There was no evidence of clinical photosensitivity in any patient. Eye closure sensitivity^25^ was reported in one patient with DOORS with generalized epilepsy (29).

Neuroimaging revealed cerebral or cerebellar atrophy in 16 patients. Five patients had delayed myelination; 3 others had hippocampal sclerosis. Eleven patients had cerebellar abnormalities: signal hyperintensity, especially in T2-weighted images (11, 14, 19, 25), atrophy (4, 5c, 5d, 7c, 10, 11, 13, 19), or mild vermian hypoplasia (29).^3,6,9,12,23,26,27^ In patient 11, progressive atrophy involved both cerebellar hemispheres, but not the vermis. There was no specific association among neuroimaging findings, phenotypic features, or prognosis.

### Developmental course.

Thirty-nine individuals had intellectual disability or developmental delay, from mild to profound. Only the 8 affected individuals of the Italian family with familial infantile myoclonic epilepsy (1a–1h) had normal psychomotor development and no signs of cognitive deterioration over an average follow-up of 52 years. Patient 2 had normal development to the most recent follow-up (aged 7 months).

### Physical examination.

Fourteen patients (29%) had DOORS syndrome (19–30). Thirty-eight patients (79%) had abnormal physical examination. The most common facial feature was a broad nasal bridge (7 individuals). Twelve individuals had cranial shape or growth abnormalities. Acral manifestations were found in 17 (35%) patients, all but 3 with DOORS syndrome. The most frequent neurologic sign was muscle hypotonia. Seven patients had ataxia. Eight patients had extrapyramidal signs (table e-3). The involvement of other organs is presented in supplemental data S2. Three patients without DOORS (17a, 17b, 18) had bilateral sensorineural hearing loss or deafness. Thirteen patients (27%), including 6 patients with DOORS, had signs of visual impairment. Patients 8a and 23b both survived an episode of cardiac arrest of unknown cause.

### Genotype–phenotype correlation.

The *TBC1D24* mutations in the 48 individuals are presented in tables e-1 and e-4. All patients had biallelic mutations, but we also include in this analysis patient 24, in whom only one *TBC1D24* mutation could be identified, but who has a typical DOORS phenotype.

**Figure 1:**
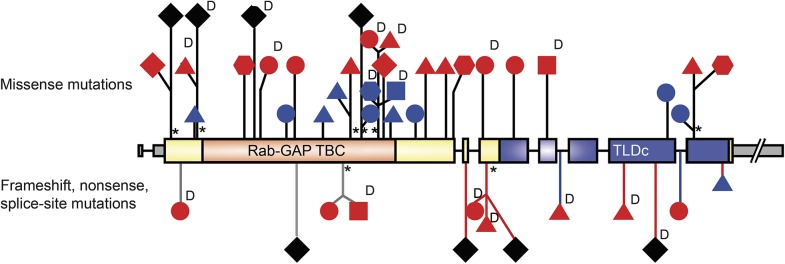
Genetic and phenotypic heterogeneity The diagram illustrates the exonic structure of *TBC1D24* isoform 1 (NM_001199107.1), with the introns as thin lines not drawn to scale. The noncoding exonic regions are drawn in gray, and the coding regions thicker and in color (orange for Rab GTPase-activating protein [Rab-GAP] domain, blue for TLDc domain, and yellow for the rest). The location of the mutations identified in various epilepsy syndromes is shown, according to the severity of the epilepsy phenotype. No clear pattern of genotype–phenotype correlation emerges. Circle = myoclonic epilepsy; square = generalized epilepsy; triangle = focal epilepsy; diamond = early-onset epileptic encephalopathy; hexagon = unclassified epilepsy. Black = death; red = drug-resistant epilepsy; blue = drug-responsive epilepsy or seizure-free. D = DOORS syndrome. Black arrows = missense mutations; red arrows = frameshift mutations; gray arrows = nonsense mutations; blue arrows = splice-site mutations; * = recurrent mutations.

Figure 1 shows the different types of mutations and the related phenotypes. We noted an unfavorable outcome associated with frameshift, nonsense, or splice-site mutations, indicating that loss of function produces more severe disease. At least one such mutation occurred in 17 patients. Of these, 15 had drug-resistant epilepsy and 8 of them died by the age of 7 years. Only the 2 siblings (12a, 12b) with focal seizures and a frameshift mutation (in the TLDc domain) in trans with a missense (p.Ala39Pro) mutation had adequate epilepsy control, following the introduction of zonisamide. The diagnosis of DOORS per se was not associated with a specific epilepsy type or outcome. No specific pattern of outcome or severity emerged in association with missense mutations, except for missense mutations associated with death: these mutations occurred in or before the TBC domain. Frameshift, nonsense, and splice-site mutations led to drug-resistant epilepsy or death, except when occurring in the last exon (figure 1). In supplemental data S3, we present genotype–phenotype correlation by mutation, rather than by patient, cross-referencing data from tables.

**Figure 2:**
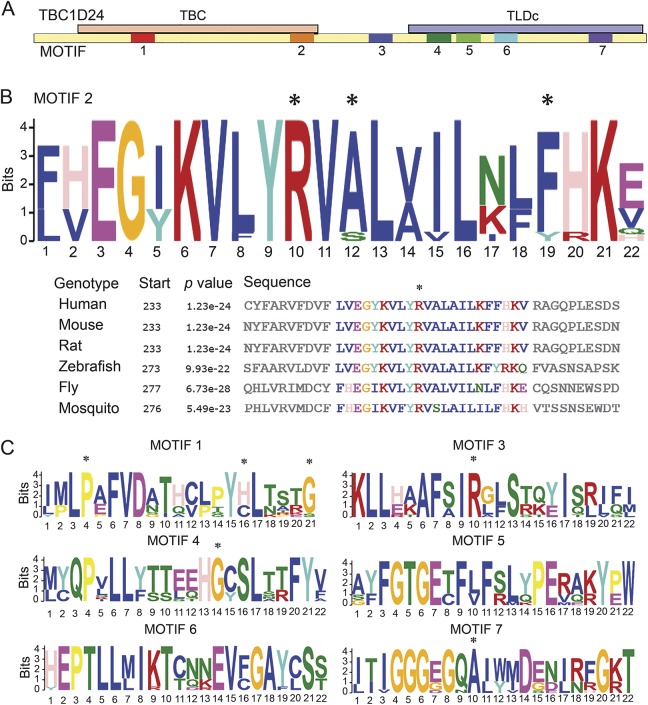
Disease-causing mutations in evolutionarily conserved motifs (A) Seven highly conserved motifs are identified in TBC1D24/Sky in an interspecies comparison against related Tre2/Bub2/Cdc16 (TBC) domain proteins. The Rab GTPase-activating protein (Rab-GAP) TBC domain is drawn in orange, the TLDc domain is drawn in blue, and the rest is in yellow, as in figure 1. (B) The consensus sequence of the TBC domain motif 2 contains an arginine residue (*) that is substituted in the most frequent pathogenic mutation seen in patients with deafness, onychodystrophy, osteodystrophy, mental retardation, and seizures. Further residues affected by pathogenic mutations are indicated by an asterisk above the residue. (C) Motif 1 in the TBC domain, motif 3 in the region between the TBC and TLDc domains, as well as motifs 4 and 7 in the TLDc domain contain further residues affected by pathogenic mutations, as indicated by an asterisk.

To identify conserved protein regions in silico, we compared human TBC1D24 and *Drosophila* Sky protein sequences with 3 vertebrate and 7 insect homologues (figure 2A). To find motifs specific for this TBC domain protein, we defined a negative set (human and mouse TBC1D1 and TBC1D7). Discriminative motif discovery yielded 7 highly conserved motifs (*E* < 10^−100^; *p* < 10^−20^) of 21–22 amino acids, completely conserved among *Drosophila* species and in key amino acids across species, signifying the importance of these domains (figure 2B). Interestingly, the second motif (figure 2B) contains the site of the most frequent mutation (p.Arg242Cys) in patients with DOORS, and the central arginine residue is conserved in every species tested. The residue altered in the p.Arg242Cys mutation is flanked by 2 lysines and pairs of valines and leucines in all sequences analyzed, suggesting that this motif's positive charge is highly conserved. In addition, motif 1 encompasses the p.Pro93Ser mutation identified in patient 9 (table e-1). Mutations also lay in motif 3, located in the region between the region between the TBC and TLDc domains, and in motifs 4 and 7, located in the TLDc domain (figure 2C), though no clear correlation with the phenotype emerges.

**Figure 3:**
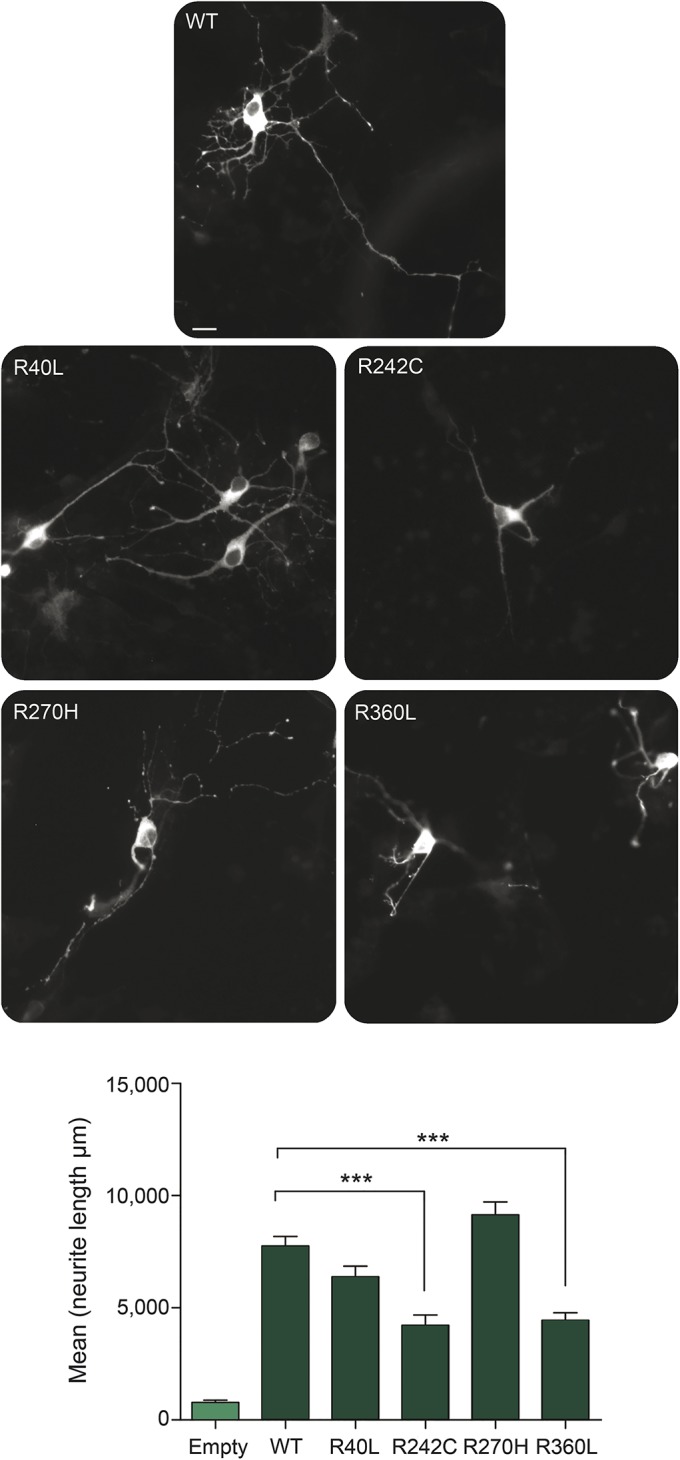
Neurite outgrowth associated with *TBC1D24* mutants Primary mouse cortical cells were transfected with wild-type (WT) or mutant *TBC1D24* constructs as indicated and representative images of transfected neurons are shown. Quantification of neurite length shows that expression of Arg242Cys (R242C) and Arg360Leu (R360L), but not Arg40Leu (R40L) or Arg270Cys (R270C), results in a significantly reduced induction of outgrowth compared to WT. ***p < 0.001, 1-way analysis of variance; scale bar = 10 μM.

To determine whether the position of the amino acid change in TBC1D24 or the corresponding severity of the disease correlate with perturbation of induction of neurite overgrowth, we studied a panel of 4 human *TBC1D24* mutants, representing the phenotypic breadth from this study: Arg40Leu,^3^ Arg242Cys,^3^ Arg270His,^23^ and Arg360Leu.^12^ In primary mouse cortical cells expressing wild-type TBC1D24, we observed a large (∼10-fold) increase in neurite outgrowth compared to those expressing a control empty vector, as expected (figure 3). All 4 TBC1D24 mutants tested were also able to promote outgrowth; however, whereas expression of either the Arg242His or Arg360Leu mutant leads to a significant reduction in neurite length compared to wild-type, the Arg40Leu and Arg270His mutants did not significantly affect outgrowth (figure 3). Together, these data indicate for the first time that mutations causing the most severe *TBC1D24*-associated disorders do not necessarily give rise to the most disruptive functional effects on neurite outgrowth in cultured neurons.

## DISCUSSION

The reported broad spectrum of epileptic and developmental phenotypes associated with *TBC1D24* mutation is unusual, and seen only with a few epilepsy-related genes, mostly with dominant causal mutations (e.g., *SCN1A*, *SCN2A*, S*CN1B*, *KCNQ2*, *KCNT1*, *PRRT2*, *DEPDC5*, *TSC1*, and *TSC2*). *TBC1D24* is associated with an even broader phenotypic spectrum, including a number of conditions other than epilepsy alone (i.e., DOORS syndrome and nonsyndromic deafness) and involving multiple organs other than the brain (see supplemental data S2). Furthermore, *TBC1D24* epilepsy syndromes occur with both compound heterozygous and homozygous recessive mutations. The additional diversity of *TBC1D24* phenotypes might be due to its broader expression pattern; *TBC1D24* is expressed in several human tissues, with the highest expression in brain, in multiple cerebral areas, including all layers of cerebral cortex and hippocampus.^1,10,28^

**Figure 4:**
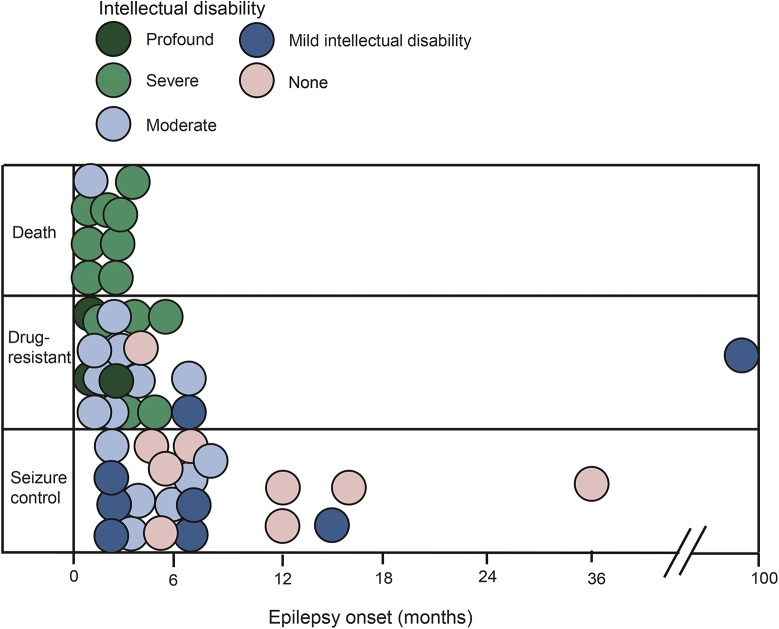
Phenotypic spectrum of patients with *TBC1D24* mutation The graph displays the age at epilepsy onset along the horizontal axis, the categorical outcome varying from seizure control to death along the vertical axis. The degree of intellectual disability is represented by the different fill of the circles, varying from white to black.

Although early-onset myoclonic epilepsy, with onset in the first year of life and myoclonic seizures often occurring in prolonged clusters, and drug resistance are the most common *TBC1D24* epilepsy phenotypes, many dissimilarities have emerged in our cohort. We delineate a spectrum ranging from a benign pattern, restricted to infancy, with complete seizure control and without intellectual disability, to early-onset epileptic encephalopathy with drug resistance, severe developmental delay, intellectual disability, and early death (figure 4). Seizure types can be diverse, often triggered by fever or infections. Episodes of status epilepticus or prolonged seizures are common. Interictal EEG and neuroimaging results are variable. There was no marked intrafamilial phenotypic variability in affected patients with the same mutations, unlike that observed in families with *SCN1A* or *DEPDC5* mutations. While we noted phenotypic pleiotropy for the same mutation in unrelated patients, in most patients these were compound heterozygous with a different second mutation, confounding detailed comparison.

Mutations leading to DOORS syndrome and those causing primarily epilepsy syndromes have mostly been mutually exclusive to date. This phenotypic pleiotropy is unexplained by current knowledge, and few genotype–phenotype correlations emerged from our study. More severe phenotypes were associated with nonsense, frameshift, or splice-site mutations, as expected, except when these occurred in the last exon. The latter might lead to milder phenotypes because RNA escapes from nonsense-mediated decay when truncating mutations occur in the ultimate exon and the end of the penultimate exon. A more variable outcome was found with missense mutations; these were associated with death only when they occurred in or before the TBC domain (figure 1). Otherwise, the occurrence of mutations in the TBC domain vs the TLDc domain did not seem to lead to different clinical phenotypes. A TLDc domain is also found in the protein encoded by the human gene *OXR1*. Mice lacking *Oxr1* display cerebellar neurodegeneration: neurons are less susceptible to oxidative stress-induced neurodegeneration when *Oxr1* is overexpressed both in vitro and in vivo.^29,30^ Not all patients with mutations in the *TBC1D24* TLDc domain in our study show clinical or neuroradiologic evidence of cerebellar neurodegeneration. The involvement of the TLDc domain in oxidative stress resistance,^20^ and thereby potentially in inflammatory mechanisms, might underlie the role of fever or infections in precipitating seizures or status in some patients. Conservation analysis showed a highly conserved arginine residue underlying the recurrent pathogenic chr16:2546873C > T transition, resulting in an arginine to cysteine (p.Arg242Cys) substitution, in the TBC domain. In the TLDc domain, 4 conserved motifs were identified. However, the analysis of evolutionarily conserved motifs also does not provide a complete answer to the diversity observed.

The phenotypic spectrum observed might partly be explained by the effect of individual *TBC1D24* mutations that affect synthesis, stability, and activity of the protein. Alternately, variations in *TBC1D24*-associated proteins and pathways could underlie the disease spectrum. The TBC1D24 protein interacts with ARF6,^1^ a small GTP-binding protein implicated in exchange between the plasma membrane and the endocytic compartments.^31^ The *Drosophila* homolog of TBC1D24, Sky, also interacts with, and activates, Rab35, a small GTPase involved in endosomal trafficking of synaptic vesicles, regulating neurotransmitter release.^2,32^ Several genetic modifiers of this pathway were identified recently.^5^ In the CNS, ARF6 participates in several aspects of neuronal development and plasticity.^33–35^ Interestingly, some pathogenic mutations in *TBC1D24* affect protein binding to ARF6 and result in severe impairment of neuronal development.^1,4,11^ Furthermore, overexpression of *TBC1D24* induces a marked increase in neurite length in vitro.^1,6^ From these initial studies, it was postulated that each of the disease-causing mutations tested (Phe251Leu, Asp147His, and Ala515Val) were likely to be loss-of-function based on the observed reduction in outgrowth compared to wild-type.^1,6^ We investigated this specific feature of TBC1D24 function for a range of pathogenic mutations and showed that mutations that cause even the most severe seizure-related disorders with neurodegeneration are not necessarily detrimental to neurite outgrowth in the chosen assay. Thus, we found no obvious correlation between this particular function of *TBC1D24* in neurons and the phenotypic spectrum we describe here. However, we provide functional data for the first time that compare pathologic human mutations in parallel across the spectrum of *TBC1D24*-associated disorders. Neuronal outgrowth has been used previously as an assay to assess TBC1D24 function, and our data extend these findings, showing that the relative position of the amino acid change in the protein, with respect to disease severity, does not correlate with abnormal outgrowth. As such, we have discovered that the association between *TBC1D24* genotype and phenotype is complex and is likely to involve several aspects of the gene's function, potentially beyond the ARF6-mediated trafficking and signaling pathways described to date.

TBC1D24 activity also regulates synaptic function. The inhibition of excessive endosomal sorting of dysfunctional synaptic vesicle proteins and their subsequent breakdown at lysosomes, by partially inhibiting the ESCRT complexes or the HOPS complex, significantly suppresses the excessive neurotransmission in *Sky* mutants.^2,5^ Variations in the aforementioned pathways may thus modify the neuronal pathway in which Sky/TBC1D24 functions, further adding to the phenotypic spectrum associated with its loss of function in patients.

Though we used a standardized template for data collection, we acknowledge that one limitation of the study is the involvement of different clinicians in defining the clinical phenotype, with potential differences in interpreting clinical, EEG, and neuroradiologic patterns. In several patients, segmental myoclonic seizures were detected on video-EEG telemetry, but had not been reported by the parents. Involvement of other systems, e.g., hearing, might also have not been formally assessed and therefore the concomitant presence of DOORS syndrome might have been underreported. We collected a modest number of patients in absolute terms, but *TBC1D24*-related epilepsy is rare and we report a relatively large series compared with other rare genetic neurologic conditions with epilepsy. We only included patients with confirmed *TBC1D24* mutations and epileptic seizures as part of their phenotype. This might represent ascertainment bias, and we may have missed individuals with neurologic involvement other than epilepsy.

Our findings support the need to test for *TBC1D24* mutation if the phenotype is appropriate. Screening should be considered, for example, if a patient has myoclonic seizures, intellectual disability, and any other extra-CNS features (including facial, cranial, acral, or other organ abnormalities).

Anyone interested in joining the TBC1D24 consortium should contact the corresponding authors.

## Supplementary Material

Data Supplement

Videos

## AUTHOR AFFILIATIONS

From the NIHR University College London Hospitals Biomedical Research Centre (S. Balestrini, S.M.S.), Department of Clinical and Experimental Epilepsy, UCL Institute of Neurology, London, and Epilepsy Society, Chalfont-St-Peter, Bucks, UK; Neuroscience Department (S. Balestrini), Polytechnic University of Marche, Ancona, Italy; Aix Marseille Université (M.M.), INSERM, GMGF UMR_S 910, Marseille, France; Child Neurology Unit (C.C.), Department of Pediatrics, Clínica Las Condes, Santiago, Chile; Laboratory of Neuronal Communication (K.L., P.V.), VIB Center for the Biology of Disease and KU Leuven Department of Human Genetics, Belgium; Department of Physiology, Anatomy and Genetics (M.J.F., P.L.O.), and National Institute for Health Research Biomedical Research Centre, Wellcome Trust Centre for Human Genetics (A.T.P., J.C.T.), University of Oxford, UK; Department of Pediatrics (A.C., J.L.H.), Division of Neurology and Developmental Neuroscience, Baylor College of Medicine, Houston, TX; Department of Child, Adolescent & Developmental Neurology (B.G.S.), University Children's Hospital, Ljubljana, Slovenia; Departments of Medical Genetics (G.L.) and Neuropediatrics (A.L.P., D.V.), Lyon University Hospital, France; Unit of Child Neuropsychiatry, Head-Neck and Neuroscience Department (M.M.M.), Pediatric Neurology and Muscular Diseases Unit, Department of Neurosciences, Rehabilitation, Ophthalmology, Genetics, Maternal and Child Health (P.S.), and Laboratory of Neurogenetics, Department of Neuroscience (F.Z.), Institute “G. Gaslini,” Genoa, Italy; Center for Genetics and Genomics (G.M. Repetto), Facultad de Medicina, Clínica Alemana, Universidad del Desarrollo, Santiago, Chile; Manchester Centre for Genomic Centre for Genetic Medicine (S. Banka), Institute of Human Development, Faculty of Medical and Human Sciences, University of Manchester, UK; Department of Neurosciences and Reproductive and Odontostomatological Sciences (L.B., S.P.), “Federico II” University, Naples, Italy; University of Wisconsin–Madison (L.E.B., G.M. Rice); Department of Neuropediatrics (F.B.), Children's Hospital Fürth, Germany; Interdisciplinary Pediatric Center for Children with Developmental Disabilities and Severe Chronic Disorders (K.B.), Faculty of Medicine, University of Göttingen, Germany; UCL Institute of Child Health & Great Ormond Street Hospital for Children NHS Foundation Trust (J.H.C.), London, UK; AP-HP (D.D.), Service de Neuropédiatrie, Hôpital Trousseau, Paris, France; Medical Genetics Service (T.M.F.), Clinical Hospital of Porto Alegre, Brazil; Department of Medical Genetics (F.G.), Nice Teaching Hospital, France; Department of Pediatrics (M.H.), Toyohashi Municipal Hospital, Toyohashi, Aichi, Japan; Institut für Humangenetik (I.H.), Universität zu Lübeck, Germany; Medical Genetics Department (H.K.), Koç University School of Medicine (KUSoM), İstanbul, Turkey; Department of Clinical Genetics (U.K.), Oxford University Hospitals NHS Trust, Oxford; North Thames Cleft Centre and Clinical Genetics Department (M.M.L.), Great Ormond Street Hospital NHS Trust, London, UK; Department of Pediatrics (G.M.), NKP Salve Institute of Medical Sciences and Lata Mangeshkar Hospital, Nagpur, India; Department of Paediatric Neurology (L.M.), Imperial College Healthcare NHS Trust, London, UK; Pediatric Neurology (A.M.), Braunschweig Hospital, Germany; Department of Molecular and Human Genetics (J.A.R., M.W.), Baylor College of Medicine, Houston, TX; Division of Pediatric Neurology (M.M.T.), Barrow Neurological Institute at Phoenix Children's Hospital, AZ; Courtagen Life Sciences Inc. (C.M.S.), Woburn, MA; Institut de Neurobiologie de la Méditerranée INSERM UMR901 (A. Falace), Marseille, France; Department of Experimental Medicine (A. Fassio), University of Genoa, Italy; Institute of Human Genetics (J.R.L.), University of Leipzig; CeGaT GmbH (S. Biskup), Tübingen, Germany; Department of Pediatrics (J.T., N.F.A., P.M.C.), Sainte-Justine Hospital, University of Montreal, Canada; Department of Molecular Biology and Genetics (A.T.), Boğaziçi University, Istanbul, Turkey; Neurogenetics Program (M.C., J.G.), School of Medicine and the Robinson Research Institute, The University of Adelaide, Australia; Sackler School of Medicine (Z.A.), Tel-Aviv University, Ramat Aviv, Israel; Department of Neurology (K.B.H., I.E.S.), Royal Children's Hospital, Melbourne, Australia; Florey Institute and Department of Pediatrics (K.B.H., I.E.S.), Royal Children's Hospital, University of Melbourne; Neurosciences (K.B.H.), Murdoch Childrens Research Institute; Epilepsy Research Centre (K.L.O., S.F.B., I.E.S.), University of Melbourne, Austin Health, Heidelberg, Australia; and Neurological Care Unit (F.A.d.F.), LoretoMare Hospital, Naples, Italy.

## GLOSSARY

ARF6: ADP ribosylation factor 6
DOORS: deafness, onychodystrophy, osteodystrophy, mental retardation, and seizures
EIMFS: epilepsy of infancy with migrating focal seizures
Rab-GAP: Rab GTPase-activating protein
TBC: Tre2/Bub2/Cdc16
