# Supplementary material for: TBC1D24 genotype–phenotype correlation: Epilepsies and other neurologic features
Source: Neurology. 2016 Jul 5;87(1):77–85. doi: 10.1212/WNL.0000000000002807 (PMC4932231; doi:10.1212/WNL.0000000000002807)
Supplement: Data Supplement [file supp_87_1_77__index.html]

TBC1D24 genotype–phenotype correlation — Data Supplement 

# *TBC1D24* genotype–phenotype correlation

## Data Supplement

**Neurology® data supplements are not copyedited before publication. Published editorials and translations have been copyedited.  
 © 2016 American Academy of Neurology.  
  
 Files in this Data Supplement:**

- Figure e-1 - PDF
- Data Supplement - Microsoft Word file
