# Supplementary material for: TBC1D24 genotype–phenotype correlation: Epilepsies and other neurologic features
Source: Neurology. 2016 Jul 5;87(1):77–85. doi: 10.1212/WNL.0000000000002807 (PMC4932231; doi:10.1212/WNL.0000000000002807)
Supplement: Data Supplement [file supp_WNL.0000000000002807_Figure_e-1.pdf]

**Figure e-1. Family History**

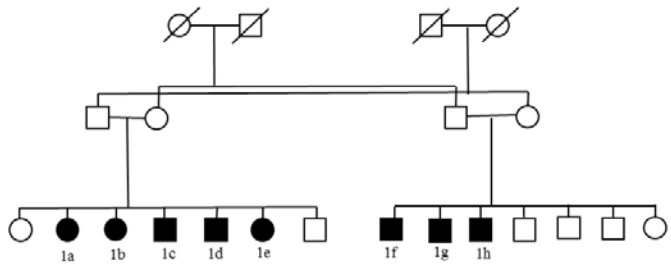


Family 1a-h

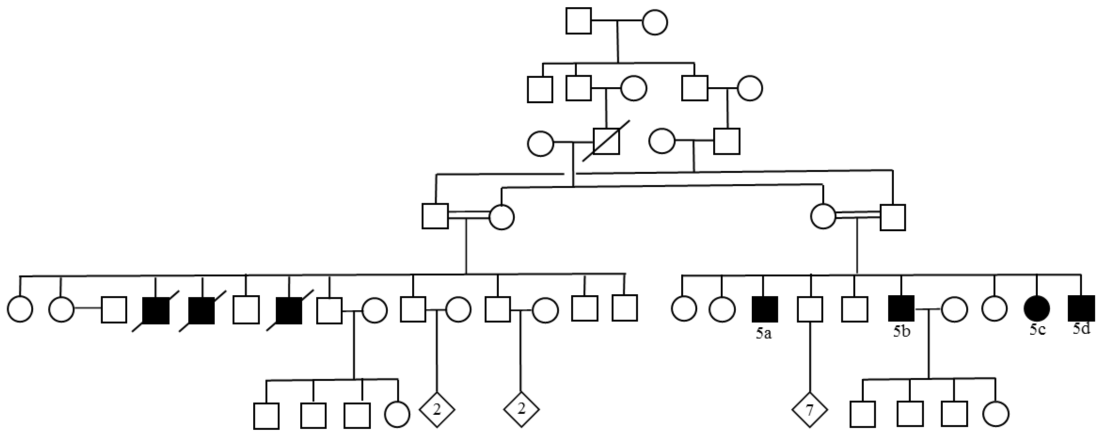


Family 5a-d

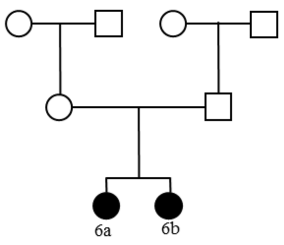


Family 6a-b

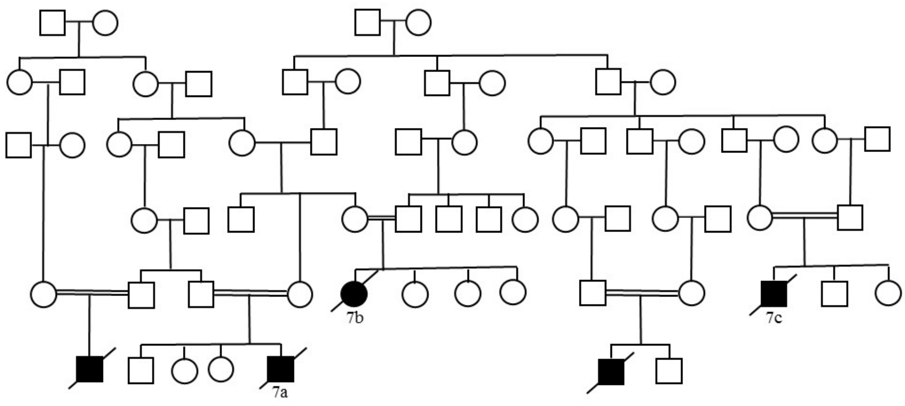


Family 7a-c

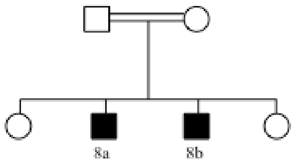


Family 8a-b

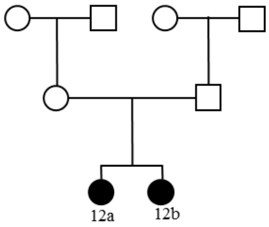


Family 12a-b

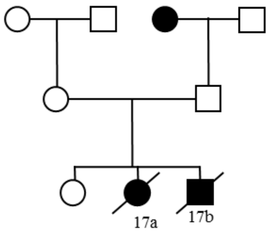


Family 17a-b

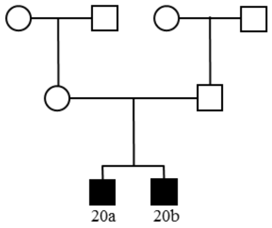


Family 20a-b

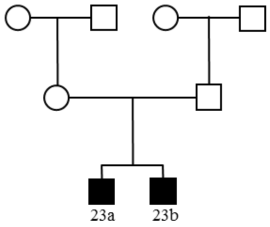


Family 23a-b
