# Supplementary material for: TBC1D24 genotype–phenotype correlation: Epilepsies and other neurologic features
Source: Neurology. 2016 Jul 5;87(1):77–85. doi: 10.1212/WNL.0000000000002807 (PMC4932231; doi:10.1212/WNL.0000000000002807)
Supplement: Data Supplement [file supp_WNL.0000000000002807_707513_Supplemental_data_amended.docx]

**Supplemental data**

**S1. Methods**

**Procedures.** *TBC1D24* variants were identified through a variety of methods, including whole exome sequencing and Sanger validation (patients #4, #6a, #7a-c, #9, #12a, #13, #16, #19, #20a, #21, #22, #23a, #26, #27), Sanger sequencing (patients #1a-h, #6b, #8a-b, #12b, #14, #20b, #23b, #24, #25, #28, #29, #30), next-generation sequencing panels (patients #2, #3, #15, #17a-b, #18, #21) and multipoint linkage analysis followed by targeted sequence enrichment and massively parallel sequencing (patients 5#a-d). The longest *TBC1D24* isoform, harbouring exon 3, was referenced (isoform 1, NM_001199107.1, GRCh37/hg19). Forty-seven patients had biallelic mutations. In only one patient (#24) was a mutation not identified in the second allele. For this patient, real-time PCR of genomic DNA for all coding exons was performed to detect a deletion or insertion of a whole exon, and the last intron was sequenced. None of these procedures identified a second mutation. The other mutation for this patient is probably in the promoter or in a deep intronic region, affecting, for example, a branch site. We did not perform further tests to explore this, since there was no clinical doubt regarding the diagnosis of DOORS syndrome.

**Bioinformatics.** TBC1D24 sequences from *H. sapiens*, *M. musculus*, *R. norvegicus* and *D. melanogaster* Sky*,* and sequences from seven other insect Sky proteins were selected as the positive set, then compared to a discriminative set of TBC-domain proteins, consisting of human and mouse TBC1D1 and TBC1D7. *E*-values and *p*-values were calculated. *E*-values estimate the likelihood of a conserved motif based on all input sequences against the amino acid composition background of the input (Sky/TBC1D24 sequence), whereas p-values estimate the probability of conservation for each protein and motif.

***In vitro* modelling.** Cortical cells from wild-type C57BL/6J P1 mouse pups were prepared and cultured as previously described.^e1^ 2 x 10^6^ cells were electroporated with 1.5 μg of each DNA construct by nucleofection (Amaxa). Cells were cultured for 4 days before fixation with 4% paraformaldehyde and staining with rat anti-HA (Roche) overnight at 4^o^C. Transfected cells were visualized using an anti-rat Alexa 459 secondary antibody (Life Technologies) and images were taken under fluorescence (Leica). A minimum of 60 neurites from transfected neurons representing each construct were measured using Image-J software (NIH). Human wild-type *TBC1D24* was generated by RT-PCR, adding an in-frame C-terminal HA-tag, followed by cloning into pcDNA3.1 (Invitrogen). *TBC1D24* mutants were generated by site-directed mutagenesis and were sequenced prior to cloning.

**Table e-1. Details of the epilepsy phenotype and mutations identified in TBC1D24. Longest isoform, harbouring exon 3, is used (isoform 1, NM_001199107.1, hg19).**

| **Patient number/ gender, current age*** | **Age at seizure onset** | **Seizure types and frequency** | **Details of Mc or clonic seizures, if present** | **Syndrome classification** | **Current AEDs** | **AEDs previously tried** | **Epilepsy outcome/ clinical evolution** | **Alleles, DNA (protein)** |
| --- | --- | --- | --- | --- | --- | --- | --- | --- |
| 1a/ W, 54 years | 4 months | GTC, occasional (12-16 years); Mc, weekly to monthly (4-12 years) | in childhood, spontaneous, erratic, bilateral, or massive, isolated or in clusters, could last for many hours, sometimes evolving into GTC; in youth, segmental, precipitated by fatigue/ drowsiness, acoustic/ light stimuli, repetitive movements | familial infantile myoclonic | VPA | none | still sporadic Mc, free from GTC | chr16:2546588G>C (p.Asp147His)/ chr16:2550823C>T (p.Ala515Val) |
| 1b/ W, 52 years | 7 months | GTC, occasional (12-16 years); Mc, weekly to monthly (4-12 years) | in childhood, spontaneous, erratic, bilateral, or massive, isolated or in clusters, could last for many hours, sometimes evolving into GTC; in youth, segmental, precipitated by fatigue/ drowsiness, acoustic/ light stimuli, repetitive movements | familial infantile myoclonic | PB | PHT | still sporadic Mc, free from GTC | chr16:2546588G>C (p.Asp147His)/ chr16:2550823C>T (p.Ala515Val) |
| 1c/ M, 50 years | 5 months | GTC, occasional (12-16 years); Mc, weekly to monthly (4-12 years) | in childhood, spontaneous, erratic, bilateral, or massive, isolated or in clusters, could last for many hours, sometimes evolving into GTC; in youth, segmental, precipitated by fatigue/ drowsiness, acoustic/ light stimuli, repetitive movements | familial infantile myoclonic | PB | PHT | still sporadic Mc, free from GTC | chr16:2546588G>C (p.Asp147His)/ chr16:2550823C>T (p.Ala515Val) |
| 1d/ M, 47 years | 5 months | GTC, occasional (12-16 years); Mc, weekly to monthly (4-12 years) | in childhood, spontaneous, erratic, bilateral, or massive, isolated or in clusters, could last for many hours, sometimes evolving into GTC; in youth, segmental, precipitated by fatigue/ drowsiness, acoustic/ light stimuli, repetitive movements | familial infantile myoclonic | PB | PHT | still sporadic Mc, free from GTC | chr16:2546588G>C (p.Asp147His)/ chr16:2550823C>T (p.Ala515Val) |
| 1e/ W, 43 years | 36 months | GTC, occasional (12-16 years); Mc, weekly to monthly (4-12 years) | in childhood, spontaneous, erratic, bilateral, or massive, isolated or in clusters, could last for many hours, sometimes evolving into GTC; in youth, segmental, precipitated by fatigue/ drowsiness, acoustic/ light stimuli, repetitive movements | familial infantile myoclonic | VPA | CBZ, PB | still sporadic Mc, free from GTC | chr16:2546588G>C (p.Asp147His)/ chr16:2550823C>T (p.Ala515Val) |
| 1f/ M, 59 years | 16 months | GTC, occasional (12-16 years); Mc, weekly to monthly (4-12 years) | in childhood, spontaneous, erratic, bilateral, or massive, isolated or in clusters, could last for many hours, sometimes evolving into GTC; in youth, segmental, precipitated by fatigue/ drowsiness, acoustic/ light stimuli, repetitive movements | familial infantile myoclonic | none | none | Mc triggered by repetitive movements or fatigue; sporadic GTC (every 2-3 years) | chr16:2546588G>C (p.Asp147His)/ chr16:2550823C>T (p.Ala515Val) |
| 1g/ M, 58 years | 12 months | GTC, occasional (12-16 years); Mc, weekly to monthly (4-12 years) | in childhood, spontaneous, erratic, bilateral, or massive, isolated or in clusters, could last for many hours, sometimes evolving into GTC; in youth, segmental, precipitated by fatigue/ drowsiness, acoustic/ light stimuli, repetitive movements | familial infantile myoclonic | none | none | Mc triggered by repetitive movements or fatigue; sporadic GTC (every 2-3 years) | chr16:2546588G>C (p.Asp147His)/ chr16:2550823C>T (p.Ala515Val) |
| 1h/ M, 56 years | 12 months | GTC, occasional (12-16 years); Mc, weekly to monthly (4-12 years) | in childhood, spontaneous, erratic, bilateral, or massive, isolated or in clusters, could last for many hours, sometimes evolving into GTC; in youth, segmental, precipitated by fatigue/ drowsiness, acoustic/ light stimuli, repetitive movements | familial infantile myoclonic | none | VPA | Mc triggered by repetitive movements or fatigue; sporadic GTC (every 2-3 years) | chr16:2546588G>C (p.Asp147His)/ chr16:2550823C>T (p.Ala515Val) |
| 2/ W, 5 months | 3.5 months | focal (motor), weekly; clonic, weekly; episodes of lip quivering with no EEG correlate, weekly | not present | focal | LEV, LTG | none | drug-resistant | chr16:2547068A>G (p.Asn307Asp)/ chr16:2546994C>G (p.Pro282Arg) |
| 3/ M, 2 years | 3 months | GTC, rare; Mc, daily | involving peri-oral region and/or limbs, rhythmic, bilateral, lasting up to 30 minutes, no loss of consciousness | infantile myoclonic | CLB, STP, VPA | PB | with STP complete control of GTC, moderate control of Mc | chr16:2546835T>C (p.Phe229Ser)/  chr16:2550465C>T (p.Ala500Val) |
| 4/ W, 15 years | 36 hours | GTC, monthly; Mc, daily; tonic, daily | prolonged, continuous runs of spontaneous Mc, both at rest and on maintaining posture; involving tongue, peri-ocular region, lower limbs, head and trunk; not markedly worsened by action; could result in falls, unable to feed self; precipitated by constipation, delayed medication or tiredness | progressive myoclonic | CBZ, VPA | CLZ, LEV, PIR, prednisolone, TPM | rarer GTCs (<1 / month); Mc daily; tonic seizures ceased at 7 years; progressive clinical deterioration  (see Video 1) | chr16:2548334G>T (p.Arg360Leu)/ chr16:2548334G>T (p.Arg360Leu) |
| 5a/ M, 46 years | 2 months | focal (prominent eye blinking, facial and limb jerking), weekly; GTC, weekly | not present | focal | CBZ, CLZ, PB | none | ongoing seizures, usually due to non-compliance | chr16:2546900T>C (p.Phe251Leu)/ chr16:2546900T>C (p.Phe251Leu) |
| 5b/ M, 40 years | 2 months | focal (prominent eye blinking, facial and limb jerking and dyscognitive), unknown initial frequency | not present | focal | PB, PHT, VPA | none | rare seizures continue despite medication | chr16:2546900T>C (p.Phe251Leu)/ chr16:2546900T>C (p.Phe251Leu) |
| 5c/ W, 33 years | 2 months | focal (prominent eye blinking, facial and limb jerking), weekly | not present | focal | CBZ, LTG | VPA | controlled on medication | chr16:2546900T>C (p.Phe251Leu)/ chr16:2546900T>C (p.Phe251Leu) |
| 5d/ M, 29 years | 2 months | focal (prominent eye blinking, facial and limb jerking and dyscognitive), weekly | not present | focal | CBZ, PHT, VPA | none | controlled on medication | chr16:2546900T>C (p.Phe251Leu)/ chr16:2546900T>C (p.Phe251Leu) |
| 6a/ W, deceased | 5 weeks | migrating clonic, focal (lateral deviation of the head and eyes, twitches of the eyelids, flushing or cyanosis of the face; or subtle seizures with cyanosis and movement arrest), daily (initial phase), almost permanent (stormy phase), clusters of several seizures per hour (late phase) | initially discontinuous, then continuous lasting several hours, migrating from one limb to another; no obvious triggers | epilepsy of infancy with migrating focal seizures (EIMFS) | deceased | CBZ, CLB, CLZ, KD, PB, PHT, ZNS | drug-resistant, death as probable consequence of chronic respiratory failure at 8 years | chr16:2546617C>A (p.Cys156*)/ chr16:2546835T>C (p.Phe229Ser) |
| 6b/ W, deceased | 4 weeks | migrating clonic, focal (lateral deviation of the head and eyes, twitches of the eyelids, flushing or cyanosis of the face; or subtle seizures with cyanosis and movement arrest), daily (initial phase) almost permanent (stormy phase), clusters of several seizures per hour (late phase) (see Video 2) | initially discontinuous, then continuous lasting several hours, migrating from one limb to another; no obvious triggers | epilepsy of infancy with migrating focal seizures (EIMFS) | deceased | CBZ, CLB, CLZ, KD, PB, PHT, ZNS | drug-resistant, death (probable SUDEP) at 18 months | chr16:2546617C>A (p.Cys156*)/ chr16:2546835T>C (p.Phe229Ser) |
| 7a/ M, deceased | 2 months | clonic, Mc, unknown initial frequency | erratic, segmental, multifocal, unilateral, migrating or alternating pseudo-rhythmic, affecting eye and peri-oral region, startle responses to auditory and tactile stimuli, duration increasing with age (up to 5-6 days), infrequent rhythmic clonic seizures | early-onset epileptic encephalopathy | deceased | CLB, CLZ, PB, TPM, VGB  VPA | drug-resistant; death due to an infection (3.5 years) | chr16:2547714-2547715delGT (p.Ser324Thrfs*3)/ chr16:2547714-2547715delGT (p.Ser324Thrfs*3) |
| 7b/ W, deceased | 3 weeks | focal (prolonged staring episodes associated with a general increase in muscle tonus and frightened facial expression, or aversion of the head and eyes to either side), Mc, unknown initial frequency | segmental, multifocal, unilateral, or generalised, pseudo-rhythmic, involving face and limbs, alternating and migrating, never occurring in sleep | early-onset epileptic encephalopathy | deceased | na | drug-resistant; death during status epilepticus associated with pulmonary infection  (3.5 years) | chr16:2547714-2547715delGT (p.Ser324Thrfs*3)/ chr16:2547714-2547715delGT (p.Ser324Thrfs*3) |
| 7c/ M, deceased | first month of life | focal (gaze deviation), Mc, spasms, unknown initial frequency | na | early-onset epileptic encephalopathy | deceased | na | drug-resistant; death due to pulmonary infection  (6.5 years) | chr16:2547714-2547715delGT (p.Ser324Thrfs*3)/ chr16:2547714-2547715delGT (p.Ser324Thrfs*3) |
| 8a/ M, 10 years | 24 hours of life | Mc, daily, in clusters | involving face and eyelids, sometimes extending to the limbs or the whole body; triggers: acoustic stimuli;  partially controlled with PHT | familial infantile myoclonic | CLZ, LEV, PHT, TPM | CBZ, LTG, PB, VPA | drug-resistant (Mc weekly) | chr16:2546606G>A (p.Glu153Lys)/ chr16:2546606G>A (p.Glu153Lys) |
| 8b/ M, 3.5 years | 2.5 months | Mc, in clusters, seizure-free intervals<15 days | long-lasting, segmental or generalized, variable topography, involving eyelids, peri-oral region, or the whole face, without loss of consciousness; triggers: feeding (facial Mc), febrile episodes;  partially controlled with PHT | familial infantile myoclonic | CLZ, PHT | na | drug-resistant | chr16:2546606G>A (p.Glu153Lys)/ chr16:2546606G>A (p.Glu153Lys) |
| 9/ W, 3 years | first day of life | focal, Mc, tonic, daily, in clusters | segmental or generalised, variable topography, involving eyelids, peri-oral region, or the whole face | early-onset epileptic encephalopathy | CLZ, KD, PIR, ZNS | CLB, CBZ, LEV, LTG, PB, PHT, TPM, VPA | drug-resistant (Mc daily) | chr16:2546426C>T (p.Pro93Ser)/ chr16:2550823C>T (p.Ala515Val) |
| 10/ W, 1 year | 2 months | clonic, Mc, sGTC, monthly | triggered by fever | early-onset epileptic encephalopathy | hydrocortisone, LEV, PB, VPA | none | drug-resistant (Mc daily, clonic and sGTCs monthly) | chr16:2546181A>G (p.Asp11Gly)/ chr16:2546181A>G (p.Asp11Gly) |
| 11/ M, 8.5 years | 7 months | Mc, twice a month | alternatively affecting eyelids, either the right or left limbs, sometimes all four limbs or the trunk, lasting from several hours to up to 2 weeks, mostly disappearing during sleep, no loss of consciousness, triggered by fever or fatigue | infantile myoclonic | TPM | BDZs, CBZ, LEV, PB, VPA, ZNS | good response to TPM  (3 month seizure-free period and now minor Mc triggered by fever) | chr16:2546958G>A (p.Arg270His)/ chr16:2546958G>A (p.Arg270His) |
| 12a/ W, 7 years | 15 months | focal (motor), sGTC, weekly | not present | focal | LEV, OXC, ZNS | none | >90% reduction in frequency on ZNS | chr16:2546264G>C (p.Ala39Pro)/ chr16:2550940-2550946del (p.Gln554Leufs*12) |
| 12b/ W, 13 years | 8 months | focal (motor), sGTC, weekly | not present | focal | LEV, ZNS | OXC | >90% reduction in frequency on ZNS | chr16:2546264G>C (p.Ala39Pro)/ chr16:2550940-2550946del (p.Gln554Leufs*12) |
| 13/ W, 12 years | 2 months | focal (eye deviation to the left, staring gaze, limb posturing), GTC, daily | not present | early-onset epileptic encephalopathy | CZP, FBM, PB | CBZ, KD, LEV, OXC, PHT, PRM, TPM | drug-resistant | chr16:2546880C>T (p.Ala244Val)/ chr16:2546880C>T (p.Ala244Val) |
| 14/ M, 14 years | 3 months | clonic, focal (multiple types including dyscognitive), sGTC, daily | na | multifocal | CLZ, TPM, VPA | CLB, LTG | drug-resistant, clinical deterioration | chr16:2546828C>T (p.Arg227Trp)/ chr16:2550823C>T (p.Ala515Val) |
| 15/ M, 8 years | 7 months | focal, Mc, sGTC, monthly | segmental, involving right or left hand, tongue or other facial parts | focal | OXC, SUL | AZM, CBZ, LEV, LTG, TPM  VPA, ZNS | free of sGTC, occasional Mc | chr16:2546682C>G (p.Ser178Trp)/  chr16:2546829G>A (p.Arg227Gln) |
| 16/ M, 9.5 years | 5 months | GTC, weekly | not present | unclassified | VPA, CLB | LTG | drug-resistant, daily GTC | chr16:2547106G>C (p.Lys319Asn)/ chr16:2547106G>C (p.Lys319Asn) |
| 17a/ W, deceased | 45 minutes after birth | clonic, Mc (mostly), spasms, tonic, daily, longest remission for 2 months | multifocal Mc, involving peri-oral region (and other facial parts) and limbs | early-onset epileptic encephalopathy | deceased | CLB, ESM, LEV, LTG, OXC, PB, PHT, TPM, VGB, VPA, ZNS, VPA | drug-resistant epilepsy, predominant Mc; died at the age of 20 months due to respiratory failure following respiratory infection | chr16:2548263delT (p.His336Glnfs*12)/  chr16:2546181A>G (p.Asp11Gly) |
| 17b/ M, deceased | 20 minutes after birth | clonic, Mc (mostly), tonic, daily after neonatal period until 13 months | multifocal, erratic, Mc, involving face (prominent eye twitching) and limbs | early-onset epileptic encephalopathy | deceased | CLB, ESM, LEV, LTG, OXC, PB, PHT, TPM | on continuous thiopental infusion after 13 months; died at the age of 24 months due to respiratory failure following respiratory infection | chr16:2548263delT (p.His336Glnfs*12)/  chr16:2546181A>G (p.Asp11Gly) |
| 18/ M, 11 years | 7 months | Mc, sGTC, weekly | triggered by fever and fatigue, sometimes evolving into prolonged GTC | infantile myoclonic | CLZ, PIR, VPA | CLB, LEV, LTG, TPM | drug-resistant (frequent Mc, sometimes evolving into sGTCs, usually prolonged) | chr16:2546768C>T (p.Gln207*)/ chr16:2550809A>G (splice-site) |
| 19/ M, 21 years | 2 months | focal, sGTC, weekly | not present | focal | CBZ, CLZ | CLB, PB, TPM, STP VPA, ZNS | drug-resistant | chr16:2546873C>T (p.Arg242Cys)/  chr16:2546267C>T (p.Arg40Cys) |
| 20a/ M, 15 years | 6 months | focal, unknown initial frequency | not present | unclassified | na | na | good control with LAC, LTG, TPM | chr16:2546873C>T (p.Arg242Cys)/ chr16:2546873C>T (p.Arg242Cys) |
| 20b/ M, 8 years | 4 months | focal, unknown initial frequency | not present | unclassified | na | na | good control with CZP, LAC, TPM | chr16:2546873C>T (p.Arg242Cys)/ chr16:2546873C>T (p.Arg242Cys) |
| 21/ W, 3 years | 6 weeks | focal, sGTC, daily | not present | focal | na | CLB, cortisone pulses, CLZ, DZP, LEV, LTG, MDZ, OXC, PB, SUL,VPA, ZNS | drug-resistant | chr16:2548263delT (p.His336Glnfs*12)/ chr16:2549421+5G>A (splice-site) |
| 22/ M, 6 years | 7 months | clonic, focal, sGTC, weekly | na | focal | CBZ, CLB, VPA | none | drug-resistant | chr16:2546873C>T (p.Arg242Cys)/ chr16:2546873C>T (p.Arg242Cys) |
| 23a/ M, 9 years | 3 months | focal, Mc, weekly | na | myoclonic | CLB, LTG, VPA | CLZ, LEV, PB, TPM | after initial control on PB and CLB, at the age of 11.5 years clinical deterioration with daily seizures and encephalopathy; very frequent Mc persisted after recovery (see Video 3) | chr16:2546207C>T (p.Gln20*)/  chr16:2546873C>T (p.Arg242Cys) |
| 23b/ M, 1 year | 3 months | focal and Mc, frequently; sGTC, two-three times per year | na | myoclonic | CLB, LEV, TPM | none | drug-resistant (see Video 4) | chr16:2546207C>T (p.Gln20*)/  chr16:2546873C>T (p.Arg242Cys) |
| 24/ W, 5 years | 3 months | clonic and Mc, weekly; focal and GTC, monthly | prolonged (several hours), erratic, involving one segment of a limb; no alteration of consciousness; triggered by fever | familial infantile myoclonic | CLZ, VPA | CBZ, LEV, STP, TPM | drug-resistant (prolonged Mc daily; clonic seizures monthly, requiring BDZ injection, see Video 5) | chr16:2548263delT (p.His336Glnfs*12)/  ? |
| 25/ M, 22 years | 7 months | absences, GTC, spasms, unknown initial frequency | not present | generalised | CLB, PHT | CBZ | seizure-free >12 months on CLB and PHT | chr16:2546873C>T (p.Arg242Cys)/ chr16:2546873C>T (p.Arg242Cys) |
| 26/ W, deceased | first day of life | focal, Mc, daily | na | early-onset epileptic encephalopathy | deceased | PB | drug-resistant, died at age of 6 months | chr16:2546268G>T (p.Arg40Leu)/ chr16:2546268G>T (p.Arg40Leu) |
| 27/ M, 4 years | 9 weeks | focal, GTC, Mc, tonic, daily | spontaneous, no obvious triggers | infantile myoclonic | LTG, PB, TPM | LEV, prednisolone, VPA | drug-resistant, currently Mc and tonic seizures weekly | chr16:2546477G>A (p.Gly110Ser)/ chr16:2548254G>T (p.Leu333Phe) |
| 28/ W, deceased | first day of life | focal (multiple types), spasms, frequency ranged from daily to monthly | not present | early-onset epileptic encephalopathy | deceased | CLZ, KD, LAC, LEV, MDZ, OXC, PB, PHT, steroids, TPM, VGB | drug-resistant epilepsy; died at 10 months | chr16:2550426dupA (p.His487Glnfs*71)/ chr16:2546462T>C (p.Cys105Arg) |
| 29/ W, 27 years | 8 years | GTC, initially sporadically, since the age of 15 years monthly | not present | generalised | LTG, OXC, PRM | LEV, TPM, VPA | drug-resistant | chr16:2546768C>T (p.Gln207*)/ chr16:2548381G>C (p.Gly376Arg) |
| 30/ M, 7 years | 1 week | focal (prolonged stiffening with eye and head deviation to one side),  Mc, sGTC, daily | na | focal | na | CLB, PB, TPM, VPA | drug-resistant | chr16:2550350del (p.Glu462Serfs*61)/ chr16:2550350del (p.Glu462Serfs*61) |
| 31/ W, 9 years | 6 years | focal (gazing, cyanosis, cramping of left hand, tremor, atonia), GTC, sGTC, daily, initially in clusters | not present | unclassified | LTG, VPA | dexamethasone pulses, LEV, OXC, TPM, VGB | drug-resistant (weekly seizure frequency at last follow-up) | chr16:2546328G>A (p.Arg60Gln)/  chr16:2546851G>A (p.=); *in cis* |
| 32/ M, 13 years | 3 years | absence, atonic, GTC, Mc, daily | spontaneous, unprovoked by triggers, previous reports of trigger by light but not documented | epilepsy with myoclonic atonic seizures | FBM, RFM, VNS | AZM, CLB, CLZ, ESM, KD, LEV, LTG, prednisolone, VPA | drug-resistant | 16p13.3 duplication of 407 Kb including, but not interrupting *TBC1D24* (2,481,289-2,888,632)x3 |

The patients are ordered as follows: patients with normal cognitive function (#1a-b-2); patients with intellectual disability but no other DOORS features (#3-13); patients with intellectual disability and acral manifestations (#14-16); patients with intellectual disability and hearing impairment (#17a-b-18); patients with DOORS syndrome (#19-30); patients where a clear association of the clinical phenotype with changes in *TBC1D24* could not be established (#31-32).

*Age is on January 2015.

Videos 1-5 are in Supplemental data.

All mutations were validated by Sanger sequencing.

^*^The histidine (His) at position 336 is substituted by a glutamine (Gln), which is followed by a frameshifted protein sequence (fs) ending with a termination codon (Ter) after 12 non-native aminoacids.

? In patient #24, no mutation was identified in the second allele.

The following patients have been reported to some degree previously: #1a-h (de Falco *et al*., 2001), #4 (Muona *et al*., 2014), #5a-d (Corbett *et al*., 2010), 6a-b (Milh *et al*., 2010), #7a-c (Güven et al., 2013), #8a-b (Poulat et al., 2015), #11(Doummar et al, 2015), #12a-b(Cardon & Holder, 2015), #17a-b(Gnidovec Stražišar *et al*., 2015), #19-27 (Campeau *et al*., 2014a), #29 (Bilo *et al*., 2014).

AEDs=anti-epileptic drugs; na=data not available; AZM=acetazolamide; BDZ=benzodiazepine; CBZ=carbamazepine; CLB=clobazam; CLZ=clonazepam; CZP= clorazepate; DZP=diazepam; ESM=ethosuximide; FBM=felbamate; GBP=gabapentin; KD=ketogenic diet; LEV=levetiracetam; LTG=lamotrigine; MDZ=midazolam; OXC=oxcarbazepine; PB=phenobarbital; PGB=pregabalin; PIR=piracetam; PHT=phenytoin; PRM=primidone; RFM=rufinamide; STP=stiripentol; SUL=sulthiame; TPM=topiramate; VGB=vigabatrin; VNS= vagus nerve stimulator; VPA=sodium valproate; ZNS=zonisamide; W=woman; M=man; GTC= tonic-clonic seizures without apparent focal onset; sGTC=tonic-clonic seizures with apparent focal onset; Mc=myoclonic seizures; SE=status epilepticus.

**Table e-2. EEG and neuroimaging results.**

| **Patient number** | **EEG** | **Photoparoxysmal response** | **Neuroimaging** |
| --- | --- | --- | --- |
| 1a | normal (age 12 years) | not present | na |
| 1b | generalized spike and wave; (age 14 years) | yes, Waltz type IV | normal (age 39 years) |
| 1c | normal (age 28 years) | not present | normal (age 32 years) |
| 1d | normal (age 18 years) | not present | normal (age 20 years) |
| 1e | normal (age 37 years) | not present | normal (age 38 years) |
| 1f | normal (age 18 years) | not present | na |
| 1g | left temporal abnormalities (age 19 years) | not present | na |
| 1h | normal background activity (age 15 years) | yes, Waltz type II-III | na |
| 2 | frontal abnormalities; one focal seizure without alteration of consciousness was recorded, with probable left hemispheric onset | not present | normal |
| 3 | normal | not present | normal |
| 4 | focal interictal epileptiform activity (at 5 months); focal epileptiform activity and bilaterally synchronous posterior quadrant fast spike and slow wave discharges, myoclonic jerks without correlated epileptiform discharges (at 16 months); generalised slowing and focal epileptiform discharges (at 2 years); focal slowing (at 3 and 5 years); intermittent bilaterally synchronous and independent spike-slow wave and polyspike-slow wave discharges, most prominent during early sleep (at 8 years); generalised epileptiform discharges increasing in sleep (at 9 years); generalised slowing and epileptiform discharges and frequent focal discharges, more frequent in sleep, no obvious EEG correlate with myoclonic jerks (at 11 years); interictal generalised spike wave and polyspike wave activity (at 15 years) | not present | cerebellar atrophy, right hippocampal sclerosis (at 14 years) |
| 5a | na | not present | na |
| 5b | 6-7 Hz low amplitude background; no paroxysmal activity (at 31 years) | not present | na |
| 5c | irregular background 7-11 Hz; bitemporal high amplitude theta; no epileptiform activity (at 24 years) | not present | right hippocampal sclerosis; cerebellar atrophy (at 28 years) |
| 5d | irregular background 6-9 Hz; some paroxysmal slow and sharp; no definite epileptiform activity (at 20 years) | not present | cerebellar atrophy (at 21 years) |
| 6a | first interictal: slow background activity, with slow waves, rare paroxysmal activity; interictal stormy phase: multifocal spikes, slow background activity; ictal: focal theta discharge followed by delta large amplitude hemispheric discharge; interictal late phase: absence of any organization, rare spikes in both temporal regions; myoclonic seizures associated with EEG abnormalities (frequency range 0.25-1 Hz) | na | birth: no structural brain abnormality; six months old: moderate brain atrophy sparing the posterior fossa |
| 6b | first interictal: slow background activity, with slow waves, rare paroxysmal activity; interictal stormy phase: multifocal spikes low background activity, rare spindles; ictal: focal migrating discharges; interictal late phase: absence of any organization, rare spikes in both temporal regions; myoclonic seizures associated with EEG abnormalities (frequency range 0.25-1 Hz) | na | one month old: no structural brain abnormality; nine months old: global brain atrophy (grey matter) sparing the posterior fossa |
| 7a | despite frequent seizures, several waking-sleep EEGs were within normal limits in the early months of the disease; a progressive slowing of the background activity and a gradual regression in the phasic elements of sleep became evident in later records, as periods of waking and sleep became less distinctive, as well as rare and isolated small spikes and multiple spikes that were predominantly in the frontal and central regions; myoclonic activity on EMG present throughout all EEGs, more prominent during the waking state, becoming more abundant over time | not present | MRI at 6 months of age showed a diffuse delay in myelination and a thin corpus callosum; brain CT at 2 years revealed diffuse atrophy with dilatation of the cerebral ventricles, subarachnoid space, and brain sulci |
| 7b | monotonous background activity composed of medium voltage and irregular slow waves within theta and delta ranges; amplitude was lower on the right hemisphere (left hemiplegia); there was ongoing phasic activity on the EMG channel, but no spikes were visible on the EEG channels; EMG was normal | not present | brain MRIs at the ages of 5 and 7 months normal; at 31 months, shortly following left-sided hemiplegia, diffuse atrophy with right predominance, especially of right hippocampus; brain SPECT at that age disclosed areas of hypoperfusion in right frontal lower and middle, right mesial and lateral temporal, and left mesial temporal areas |
| 7c | early EEGs were reported to have generalized and multifocal multiple spikes as well as spike-waves discharges | not present | brain MRI at the ages of 14 and 37 months revealed progressive, diffuse cerebral and cerebellar atrophy with dilatation of the ventricles, sulci, and subarachnoid space |
| 8a | always subnormal; at last follow-up (10 years), moderate and bilateral slowing of the background rhythm, 6-Hz theta and slow spikes at the vertex, as well as spike and wave activity in central regions | not present | at 11 months, normal brain MRI scan; at 6 years, after cardiac arrest, signal hyperintensity of the lentiform nuclei, moderate ventricular dilatation, and white matter rarefaction, on the T2 and FLAIR sequences |
| 8b | mostly normal; 4 months: one recorded episode of myoclonia with very low amplitude spikes, mostly at the vertex, followed by hemispheric slow waves, predominating in central regions, intermixed with rare spikes and waves; 3.5 years: rare posterior slow spikes and increased amplitude of the posterior regions, during drowsiness | not present | at the age of 3 and 23 months, normal brain MRI |
| 9 | at 1.5 months, slow rhythms with transient burst-suppression pattern; later abnormal background activity with delta slow waves, associated with spike and wave activity, more often with occipital localisation; last record showed very attenuated background activity | not present | global brain atrophy, in particular atrophy of the caudate and lenticular nuclei (8 months) |
| 10 | delta rhythm with multifocal paroxysms, no clear correlate of myoclonus (at 8 months) | na | brain atrophy (at 8 and 14 months) |
| 11 | first EEG normal (7 months); later, interictal: isolated or bursts of spikes over bifrontal regions and slow posterior waves; ictal: no paroxysmal anomalies during myoclonic jerks, but video recording with EEG jerk-locked back-averaging was  suggestive of cortical origin of myoclonus | na | at 3 and 7 years: progressive hemispheric (but not vermian) cerebellar atrophy with hyperintense signal of the cerebellar cortex and white matter, on T2 and FLAIR sequences |
| 12a | no ictal activity captured; at 4 years, slightly slow occipital dominant rhythm of 7.5 Hz without focal findings | not present | normal (23 months) |
| 12b | no ictal activity captured; at 10 years, dominant slow (7Hz) occipital rhythm, bilateral frontocentral rhythmic slow high-voltage activity | not present | normal (7 years) |
| 13 | normal at 4 months; very abnormal EEG at 6 months with paroxysmal epileptiform discharges, bouts of intense crying considered ictal on EEG | na | elevated glutamine peak (MRI);  cerebellar atrophy, volume loss in left frontal lobe, enlargement of temporal horns suggestive of bilateral hippocampal atrophy (CT) |
| 14 | interictal multifocal independent spike waves (at 13 years) | na | right hippocampal sclerosis, bilateral cerebellar atrophy, hyperintense signal of the cerebellar cortex (at 9 years) |
| 15 | mostly normal ictal and interictal EEG, rare interictal epileptic discharges fronto-centrally; in focal status epilepticus, rare seizures patterns with repetitive fronto-central epileptic discharges | not present | normal |
| 16 | excessively rhythmic background activity over parasagittal electrodes and bursts of high-amplitude slow waves anteriorly | na | normal |
| 17a | first interictal EEG recording (on first day of life) unremarkable despite frequent seizures; later ictal EEG showed generalized spike-wave and poly-spike discharges with fronto-central predominance; on several video-EEGs with myoclonic jerks, no clear epileptiform discharges were recorded; progression to burst-suppression before death | not present | unremarkable (age 1 month and 3 months) |
| 17b | first interictal EEG recording (on first day of life) unremarkable despite frequent seizures; later generalized spike-wave and multiple spike-wave discharges with fronto-central predominance, slowing of the baseline activity and multifocal spikes | not present | prominent fronto-temporal atrophy with widening of the subarachnoid spaces and Sylvian fissures (1 month) |
| 18 | no paroxysmal abnormalities until the age of 7 years, then rare bilateral sharp-waves prevalent in the right temporo-occipital regions | na | wide cisterna magna, thin corpus callosum, mild signal hyperintensity in tegmental area (at 6 years) |
| 19 | left frontal dominant spike and wave | na | thin cerebellar cortex, hyperintense signal on T2-imaging, and myelination delay - white matter damage |
| 20a | na | na | normal |
| 20b | na | na | punctate foci of increased T2 signal in right frontal region; increased FLAIR signal around occipital horn |
| 21 | normal | not present | delayed myelination (at 33 months) |
| 22 | normal | not present | normal (at 2.5 years) |
| 23a | normal EEG at the age of 10 years 7 months; poor organization, generalized slowing, frequent multifocal epileptiform activity at the age of 11 years | na | normal |
| 23b | at the age of 10 months, severely abnormal background EEG, no evident epileptiform activity | na | normal initially; at the age of 17 months, after acute hypoxic episode, marked increase in ventricular and subarachnoid space, with decrease in grey and white matter, diffuse but with occipital predominance |
| 24 | normal interictal | na | normal |
| 25 | poorly organised background with slow waves (age 18 and 19 years) | na | hyperintense T2 signals in the cerebellar hemispheres, especially on the left |
| 26 | na | na | normal cranial ultrasound after birth |
| 27 | excess of irregular slow wave activity, often of higher amplitude on the right, with occasional runs of more prominent slow wave activity over the right hemisphere, more marked posteriorly; no epileptiform activity recorded (age 7 months) | not present | bilateral cerebral atrophy, particularly of the frontal lobes with widening of the Sylvian fissures, significantly delayed myelination and thin corpus callosum (age 7 months) |
| 28 | multifocal interictal epileptiform discharges (sharp waves, fast activity, spikes, polyspikes), more frequent in sleep, disorganised and slow background, between 6 weeks and 8 months of age; ictal: spasms and multiple types of focal seizures (right temporal, left temporal, midline-left central) were recorded | na | day 7: normal; day 56: increased T2 signal in left hippocampus, prominent extra-axial cerebrospinal fluid spaces |
| 29 | dominant symmetrical monomorphic theta activity associated with rare symmetrical alpha activity and bilateral occipital paroxysmal spike-and-wave activity on eye closure (eye closure sensitivity)^e2^ (age 23 years) | not present | mild hypoplasia of the cerebellar vermis (age 23 years) |
| 30 | interictal: widespread slow activity with frequent sharp waves over mid-parietal and right parietal regions and independent brief episodes of repetitive spikes over both fronto-central regions; ictal: repetitive spikes over the left temporal leads | na | normal antenatal brain MRI; evidence of trigonocephaly due to premature fusion of the metopic suture; mild lack of white matter bulk with delayed myelination (age 4 months) |
| 31 | at onset (6years) generalized slowing of background activity, reminiscent of encephalitis; follow-up: multifocal spike-waves, bi-frontal sharp-waves, generalized sharp-waves | not present | normal (age 6 years and 7 years) |
| 32 | frequent epileptiform discharges, both hemispheres, frequently associated with eyelid jerks | photic stimulation between 4 and 8Hz provoked increasing duration of generalised spike and slow waves without clinical accompaniments | normal |

na=data not available

**Table e-3. Family history, facial and cranial features, acral manifestations and neurological examination.**

| **Patient number** | **Family history of epilepsy*** | **DOORS syndrome** | **Facial and cranial features** | **Acral manifestations** | **Other findings on neurological examination** |
| --- | --- | --- | --- | --- | --- |
| 1a-h | all siblings or double first cousins | no | none | none | none |
| 2 | older brother with epilepsy | no | none | none | none |
| 3 | none | no | thick lips, everted lower lip | none | hypotonia, nystagmus, tongue fasciculations |
| 4 | consanguineous parents | no | none | none | ataxia, bilateral spasticity, supranuclear gaze palsy, dystonia, superimposed tremor, progressive deterioration of gait (see Supplementary Video 1) |
| 5a | consanguineous parents, three siblings (5b,c,d) and three first cousins | no | none | none | dysarthria and ataxia |
| 5b | consanguineous parents, three siblings (5a,c,d) and three first cousins | no | none | none | dysarthria and ataxia, jerky pursuit eye movement |
| 5c | consanguineous parents, three siblings (5a,b,d) and three first cousins | no | none | none | jerky pursuit eye movement and mild dysarthria |
| 5d | consanguineous parents, three siblings (5a,b,c) and three first cousins | no | none | none | ataxia, jerky pursuit eye movement and increased jaw jerk |
| 6a | sister (6b) | no | acquired microcephaly | na | normal at the initial phase; loss of eye contact at 4 months, severe axial hypotonia, dystonic movements |
| 6b | sister (6a) | no | acquired microcephaly | na | normal at the initial phase; axial hypotonia from 3 months, loss of eye contact at 4 months, severe  hypotonia at 4.5 months old |
| 7a | four similarly affected relatives, all born to consanguineous parents | no | na | na | dystonic episodes (both focal and axial) from the second year of life; right-sided spastic hemiparesis as a consequence of a prolonged unilateral seizure evident at 18 months; axial hypotonia and spasticity in the extremities with asymmetrical pyramidal signs were detected at the age of 21 months; at the age of 23 months opisthotonic posturing was predominant, and repetitive startle responses occurred, without habituation |
| 7b | four similarly affected relatives, all born to consanguineous parents | no | na | na | at the age of 31 months, left- sided epilepsia partialis continua started, associated with a permanent hemiplegia; two months later, she was inattentive and only partly responsive to verbal stimuli; there was spastic hemiparesis and dystonia on the left side |
| 7c | four similarly affected relatives, all born to consanguineous parents | no | na | na | at the age of 3 years, totally nonreactive to all environmental stimuli; generalized hypotonia with pyramidal signs and minor erratic myoclonia; pupils dilated, with no light response |
| 8a | brother (8b) | no | acquired microcephaly; bulbous nasal tip, flat nasal root | none | at seizure onset (24 hours of life), global hypertonia and incessant crying; at last follow-up (10 years), spastic tetraparesis, no verbal language |
| 8b | brother (8a) | no | bulbous nasal tip, flat nasal root, down-slanting palpebral fissures | none | at 3.5 years, he could walk with help and say 10 words |
| 9 | none | no | bulbar nose, flat nasal root | none | pyramidal tract signs |
| 10 | no | no | microcephaly | none | none |
| 11 | none | no | narrow forehead, thin upper lip, marked philtrum | none | mild ataxia, clumsiness and tremor |
| 12a | sister (12b) | no | upslanting palpebral fissures, increased intercanthal distance | none | none |
| 12b | sister (12a) | no | none | none | none |
| 13 | none | no | microcephaly, widely spaced teeth | none | choreoatethoid movement, dystonia, hypotonia, spastic quadriplegia |
| 14 | none | no | triangular face | onychodystrophy in 4th and 5^th^ toenail | ataxia, hand tremor, progressive gait deterioration, no verbal language |
| 15 | paternal great uncle | no | relatively large round head, smaller right eye | small hands and short fat fingers | fine motor deficits, slight balance problems |
| 16 | none | no | small head, large ears, narrow palpebral fissures, prominent nasal bridge, protruding nasal tip, prominent incisors, microcephaly | clinodactyly of fourth and fifth toes | unable to walk |
| 17a | paternal grandmother, father’s half-sister and brother (17b) | no | none | none | axial hypotonia, dyskinetic movements with upper limb dystonia |
| 17b | paternal grandmother, father’s half-sister and sister (17a) | no | acquired microcephaly | none | axial hypotonia, dyskinetic movements with upper limb dystonia |
| 18 | two cousins in paternal line with epilepsy | no | none | none | mild ataxia, severe hyperactivity |
| 19 | none | yes | prominent eyelashes, thick eyebrows, narrow palpebral fissures, thick lower vermilion | small nails all fingers and toes bilaterally, hand brachydactyly, triphalangeal thumb, long thumbs and halluces | none |
| 20a | brother with DOORS syndrome (20b) | yes | large central incisors, widely spaced teeth, delayed eruption of permanent teeth, sagittal craniosynostosis | hand and foot abnormalities (involving nails and fingers/toes), hand brachydactyly, calcaneal deformity | na |
| 20b | brother with DOORS syndrome (20a) | yes | na | hand and foot abnormalities (involving nails and fingers/toes), hand brachydactyly | na |
| 21 | na | yes | microcephaly | hand and foot abnormalities (involving nails and fingers/toes), hand brachydactyly | at 5 years, could say a few words, use sign language, broad-based gait |
| 22 | one sibling with epilepsy | yes | low anterior hairline, thick hair, narrow forehead, prominent eyelashes, synophrys, thick eyebrows, broad nasal bridge, stained teeth | hand and foot abnormalities (involving nails and fingers/toes), hand brachydactyly, triphalangeal thumb, long thumbs and halluces, hypoplastic distal phalanx, fetal finger pads | none |
| 23a | brother with DOORS syndrome (23b) | yes | prominent nose and ears, widely spaced teeth, brachycephaly | hand and foot abnormalities (involving nails and fingers/toes), hand brachydactyly | none |
| 23b | brother with DOORS syndrome (23a) | yes | prominent nose and ears, widely spaced teeth | hand and foot abnormalities (involving nails and fingers/toes), hand brachydactyly | hypotonia, absent eye contact |
| 24 | mother | yes | microcephaly, bilateral epicanthus, thin upper lip, reverse dental articulation, moderate mid face retraction | hand and foot abnormalities (involving nails and fingers/toes), hand brachydactyly | hypotonia, ataxia |
| 25 | none | yes | thick eyebrows, downward slant palpebral fissures, wide nasal bridge, thick alae nasi, broad nasal bridge | hand brachydactyly, small 5^th^ finger with hypoplastic distal phalanx, small nails all fingers and toes | none |
| 26 | similarly affected brother, who died at the age of 9 months; consanguineous parents | yes | broad nasal tip, narrow palate, prominent and broad alveolar ridge, sparse eyebrows, long and prominent philtrum, thin upper lip, short lingual frenulum causing furrowing of the tongue, capillary hemangioma (glabella and at nose), mild bitemporal narrowing, flat glabella, prominent occiput, parietal prominence, frontal bossing wide fontanelles | hand brachydactyly, triphalangeal thumb | hypotonia |
| 27 | none | yes | broad nasal tip, thick alae nasi, broad nasal bridge, broad and longish philtrum; thick lower vermillion, drooping lower lip, thick hair at 3 years and 7 months, high arched palate, asymmetric brachycephaly | hand brachydactyly, long halluces, small 5^th^ finger, absent distal phalanx 5^th^ finger; fetal finger pads; bilateral small nails all fingers/toes | hypotonia, congenital |
| 28 | father | yes | premature closure of fontanelle, coarse facial features | absent toenail and end of the right second toe | hypotonia, nystagmoid eye movements |
| 29 | none | yes | frontal bossing, sunken nasal bridge, low-implanted ears | anonychia on the first and fifth finger of both hands and on all toes, presence of small dystrophic nails on the remaining fingers, absence of the distal phalanx of the fifth finger of both hands and hypoplasia of the distal phalanxes of the remaining fingers | parkinsonism with onset at 21 years (mixed resting-postural tremor and rigidity of the right arm and right-side bradykinesia; mild dysdiadochokinesia and reduced tendon reflexes |
| 30 | one sibling affected prenatal (pregnancy terminated because of increased nuchal of 5.1mm at 12/40) | yes | arched, fine eyebrows; bilateral pre-auricular tags, narrow ear canals; broad nasal bridge; cleft lip and alveolus; gum hypertrophy, bifid uvula; metopic ridging, leading to appearance of trigonocephaly, microcephaly | absent distal phalanges of fingers from index to little in both hands, with hypoplastic distal phalanx of thumbs; in feet, absent distal phalanges all toes, with hypoplastic middle phalanges of little toes; stippling of tarsal bones; absent nails of all digits on hands and feet | initially hypotonia, then developed hypertonia; no speech; unable to walk |
| 31 | none | no | broad nasal bridge, high arched palate | small toenails | mild hypotonia, no focal neurological abnormality |
| 32 | none | no | none | na | none |

na=data not available

*There were seven similarly affected siblings (one in family 2, three in family 5, one in family 7, one in family 22 and one in family 27) that did not have DNA analysis and were not included in our analysis, but we presume they had the same mutation(s) as their siblings. We thus identified two more patients (one affected patient in family 7, and a sibling of patient #27) with myoclonic epilepsy and presumed *TBC1D24* mutation. Overall, with these additional patients, clonic or myoclonic seizures were present in 32/55 (58%) of patients.

**S2. Multi-organ involvement.**

The following non-DOORS patients had hearing loss: #17a (bilateral sensorineural hearing loss of 50 dB threshold), #17b (profound sensorineural deafness), #18 (bilateral hypoacusia). There was family history of hearing loss in patients #16 (brother) and #31 (hearing loss in maternal grandmother from childhood onwards, now nearly deaf).

There was visual impairment in patients: #3 (nystagmus), #4 (nystagmus, visual decline), #6a (pre-terminal cortical blindness), #7c (bilateral optic atrophy and macular degeneration), #10 (no ocular pursuit, severe encephalopathy), #15 (+2 dioptres in the left eye), #20a-b and 21 (all with myopia), #23a, #23b (post- asphyxia), #28 (cortical visual impairment), #30.

Renal anomalies were present in two patients: #21 (nephrocalcinosis) and #27 (hydronephrosis left kidney, prenatal diagnosis).

Skeletal anomalies, other than acral manifestation, were found in patients: #4 (bilateral cavovarus deformities; significant external tibial torsion; kyphotic posture), #6a-b, 13 and 30 (all with scoliosis).

Cardiac anomalies were present in patients: #8a (at 6 years and 10 months, cardiac arrest of unknown cause), #20b (double outlet right ventricle), #23b (at 10 months, cardiac arrest of unknown cause), #28 (patent foramen ovale).

Feeding difficulties were reported in patients: #4 (due to myoclonus), #6a-b (both with gastrostomy), #7a-c, #9 (enteral feeding), #10, #11 (only during episodes of facial myoclonus), #13 (naso-gastric tube from 3 years), #16, #17a (gastrostomy), #17b (naso-gastric tube), #20a-b, #23a-b, #28, #30 (naso-gastric tube from 4 months).

Other co-morbidities were described in patients; #4(severe iron deficiency, severe constipation, drooling, cataplexy, neonatal jaundice), #7a-c (repeated episodes of common infections), #8a (self-injurious behaviour and sleep disturbance), #13 (paracentric inversion (1)(q42.13q44) on karyotype; Raynaud phenomenon in lower extremities), #16 (motor neuropathy), #19 (autism spectrum disorder), #24 (hyperactivity, sleep disorder), #25 (hypothyroidism), #27 (cryptorchidism, frequent respiratory tract infections), #28 (periods of irregular breathing in wake; irritability; poor sleep), #29 (supernumerary nipple; asymptomatic peripheral polyneuropathy, mixed axonal-demyelinating sensorimotor; psychotic symptoms).

Urine 2-oxoglutarate was elevated in patients #4, #15, #20b, #25, #26, #27, #28, #29.

**Table e-4. Coding DNA position and ExAC allele count of the mutations identified in *TBC1D24*. Longest isoform, harbouring exon 3, is used (isoform 1, NM_001199107.1, hg19).**

| **Patient number** | **Origin** | **Alleles, DNA (protein)** | **ExAC allele count**  **(version 0.3, accessed 18.10.15)** | **Reference** |
| --- | --- | --- | --- | --- |
| 1a-h | Italy | c.439G>C (p.Asp147His)/ c.1544C>T(Ala515Val) | not present/  3/103786 | de Falco et al., 2001 |
| 2 | USA | c.919A>G(p.Asn307Asp)/ c.845C>G(p.Pro282Arg) | not present/  21/120442 | unreported |
| 3 | Germany | c.686T>C(p.Phe229Ser)/ c.1499C>T(p.Ala500Val) | not present/  not present | unreported |
| 4 | Afghanistan | c.1079G>T(p.Arg360Leu)/ c.1079G>T(p.Arg360Leu) | 1 / 84934 | Muona et al., 2014 |
| 5a-d | Arab-Israeli family | c.751T>C(p.Phe251Leu)/ c.751T>C(p.Phe251Leu) | not present | Corbett et al., 2010 |
| 6a-b | France | c.468C>A(p.Cys156*)/ c.686T>C (p.Phe229Ser) | not present/  not present | Milh et al., 2013 |
| 7a-c | Turkey | c.969_970delGT (p. Ser324Thrfs*3)/ c.969_970delGT (p. Ser324Thrfs*3) | not present | Güven et al., 2013 |
| 8a-b | France | c.457G>A p.Glu153Lys)/ c.457G>A p.Glu153Lys) | 10 / 118188 | Poulat et al., 2015 |
| 9 | France | c.277C>T(p.Pro93Ser)/ c.1544C>T(p.Ala515Val) | not present/  3 / 103786 | unreported |
| 10 | Italy | c.32A>G(p.Asp11Gly)/ c.32A>G(p.Asp11Gly) | not present | unreported |
| 11 | France | c.809G>A(p.Arg270His)/ c.809G>A(p.Arg270His) | 2 / 120532 | Doummar et al, 2015 |
| 12a-b | USA | c.115G>C(p.Ala39Pro)/ c.1661_1667del(p.Gln 554Leu fs*12) | not present/  not present | Cardon & Holder, 2015 |
| 13 | USA | c.731C>T(p.Ala244Val)/ c.731C>T(p.Ala244Val) | 2 / 120538 | unreported |
| 14 | Chile | c.679C>T(p.Arg227Trp)/ c.1544C>T(p.Ala515Val) | 2 / 120400/  3 / 103786 | unreported |
| 15 | Germany | c.533C>G(p.Ser178Trp)/ c.680G>A(p.Arg227Gln) | not present/  3 / 120392 | unreported |
| 16 | Pakistan | c.957G>C(p.Lys319Asn)/ c.957G>C(p.Lys319Asn) | not present | unreported |
| 17a-b | Slovenia | c.1008delT(p.His336Glnfs*12)/ c.32A>G(p.Asp11Gly) | not present/  not present | Gnidovec Stražišar et al., 2015 |
| 18 | Italy | c.619C>T(p.Gln207*)/ c.1530A>G(splice-site) | 3 / 120282/  2 / 95480 | unreported |
| 19 | Japan | c.724C>T(p.Arg242Cys)/ c.118C>T(p.Arg40Cys) | 1 / 120504/  2 / 119952 | Campeau et al., 2014a |
| 20a-b | USA | c.724C>T(p.Arg242Cys)/ c.724C>T(p.Arg242Cys) | 1 / 120504 | Campeau et al., 2014a |
| 21 | Germany | c.1008delT(p.His336Glnfs*12)/ c.1206+5G>A(spice-site) | not present/  not present | Campeau et al., 2014a |
| 22 | India | c.724C>T(p.Arg242Cys)/ c.724C>T(p.Arg242Cys) | 1 / 120504 | Campeau et al., 2014a |
| 23a-b | Chile | c.58C>T(p.Gln20*)/ c.724C>T(p.Arg242Cys) | 10 / 120240/  1 / 120504 | Campeau et al., 2014a |
| 24 | France | c.1008delT(p.His336Glnfs*12)/ not identified | not present | Campeau et al., 2014a |
| 25 | Brazil | c.724C>T(p.Arg242Cys)/ c.724C>T(p.Arg242Cys) | 1 / 120504 | Campeau et al., 2014a |
| 26 | Turkey | c.119G>T(p.Arg40Leu)/ c.119G>T(p.Arg40Leu) | not present | Campeau et al., 2014a |
| 27 | UK | c.328G>A(p.Gly110Ser)/ c.999G>T(p.Leu333Phe) | 1 / 118678/  not present | Campeau et al., 2014a |
| 28 | Australia | c.1460dupA(p.His487Glnfs*71)/c.313T>C(p.Cys105Arg) | not present/  not present | unreported |
| 29 | Italy | c.619C>T(p.Gln207*)/ c.1126G>C(p.Gly376Arg) | 3 / 120282/  not present | Bilo et al., 2014 |
| 30 | Afghanistan | c.1384del(p.Glu462Serfs*61)/ c.1384del(p.Glu462Serfs*61) | not present | unreported |
| 31 | Germany | c.179G>A(p.Arg60Gln)/ c.702G>A(p.=); *in cis* | 26 / 119704/  23 / 120454 | unreported |
| 32 | UK | 16p13.3 duplication of 407 Kb including, but not interrupting, *TBC1D24* (2,481,289-2,888,632)x3 | / | unreported |

All mutations were validated by Sanger sequencing. Individuals with letters a-h after the number are part of families.

^*^The histidine (His) at position 336 is substituted by a glutamine (Gln), which is followed by a frameshifted protein sequence (fs) ending with a termination codon (Ter) after 12 non-native aminoacids.

? In patient #24, no mutation was identified in the second allele.

The ExAC frequency was not matched for ethnicity.

Patients #31 and #32 were not included in the final analysis because a clear association of the clinical phenotype with changes in *TBC1D24* could not be established.

**S3. Genotype-phenotype correlation**

Below, details are provided about the most recurrent mutations, presented in order of frequency.

The most common recurrent mutation was chr16:2546873C>T transition (hg19 numbering used), resulting in an arginine to cysteine (p.Arg242Cys) substitution in the TBC domain, present in seven individuals in five unrelated families of different national origins (Japan, USA, India, Chile and Brazil), all with DOORS syndrome.^e3^ Four individuals were homozygous (#20a-b; #22; #25) for this missense variant, and three were compound heterozygous (#19; #23a-b). Three patients had different epilepsy types and ‘adequate’ seizure control; one patient (#25) was seizure-free on phenytoin and clobazam. Patients #19, #22 and #23b were drug-resistant. Patient #23a, after initial satisfactory seizure control on phenobarbital and clobazam, at the age of 11.5 years showed clinical deterioration with daily seizures and encephalopathy. He was treated with a combination of clobazam, lamotrigine and sodium valproate, with gradual resolution of the encephalopathy, after which he continued to have very frequent myoclonic movements (not rhythmic, rather erratic, sometimes ameliorating with postural changes) of the arms, sometimes also affecting the legs, without impairment of awareness (see Supplemental Video 2). The degree of intellectual disability varied from mild to severe. The interictal EEG showed different patterns: focal abnormalities (#19), normal (#22 and initially for #23a), poor organization, generalised slowing and multifocal abnormalities (later #23a), poor organization without epileptiform activity (#23b), and poor organization and slow waves (#25).

The frameshift mutation chr16:2548263delT (p.His336Glnfs*12) was identified as part of compound heterozygosity in four patients (#17a-b; #21 and #24; in the last patient no mutation was detected in the other allele). This mutation was not in a known functional domain. All these four patients had severe drug-resistant epilepsy with early seizure onset (ranging from 45 minutes after birth to three months of age) and multiple seizure types. Three have microcephaly and/or hypotonia. All have profound bilateral sensorineural hearing loss. These patients are two unrelated patients with DOORS syndrome and one sibling pair with early-onset epileptic encephalopathy and early death.

The heterozygous missense chr16:2550823C>T (p.Ala515Val) mutation, in the TLDc domain, was present in the eight members (#1a-h) of the Italian family with familial infantile myoclonic epilepsy, in a French patient with early-onset epileptic encephalopathy (#9) and in a Chilean patient (#14) with multifocal epilepsy. The epilepsy type and outcome were quite different, varying from myoclonic or tonic-clonic seizures, well-controlled on one or no antiepileptic medication (patients #1a-h), to myoclonic, clonic, focal or tonic-clonic seizures with focal onset, not responsive to antiepileptic treatment (patients #9 and #14). Patients #1a-h had no signs of cognitive impairment and a normal neurological examination, while patients #9 and #14 had severe to profound intellectual disability and neurological abnormalities. Functional experiments suggest that this variant causes a loss of function of TBC1D24 protein.^e4^

The heterozygous missense chr16:2546835T>C (p.Phe229Ser) mutation, in the TBC domain, was found in three individuals (#3; #6a-b), one with infantile myoclonic epilepsy and two siblings with familial epilepsy of infancy with migrating focal seizures (EIMFS). Functional assay revealed loss of ARF6 binding.^e5^ Clonic or myoclonic seizures, and hypotonia, were reported in all three patients. Neuroimaging was initially unremarkable, but in the two siblings later showed supratentorial brain atrophy in the two siblings. The two siblings had a more severe phenotype and early death.

The missense mutation chr16:2546181A>G (p.Asp11Gly) is not in a known functional domain, and was detected in three individuals (#10; #17a-b), all with early-onset epileptic encephalopathy. Of these, two were a sibling pair who had the mutation as part of compound heterozygosity in combination with the above-described frameshift mutation chr16:2548263delT, while the other individual was homozygous. All three patients had drug-resistant epilepsy, predominantly myoclonic seizures, and severe intellectual disability. EEG showed multifocal epileptiform activity. Brain MRI scan (all performed between 1 to 16 months of age) revealed brain atrophy in two patients (#10, #17b) and was normal in one other (#17a). Microcephaly was reported in patients #10 and #17b. The two siblings #17a and #17b both had profound hearing loss and died early.

Another recurrent heterozygous mutation in the TBC domain was the transition chr16:2546768C>T, leading to a premature termination codon, p.Gln207*. This mutation was found in two unrelated patients (#18; #29), one with generalised epilepsy, DOORS syndrome and parkinsonism^e6^ and the other with infantile myoclonic epilepsy. Both patients were drug-resistant, with tonic-clonic seizures. Both had bilateral hypoacusia/deafness, but patient #18 did not have onychodystrophy or osteodystrophy and was therefore not classified as DOORS syndrome.

We report two additional patients (#31, #32) with variants in *TBC1D24* of uncertain significance for the clinical phenotype (Tables e1-4). Patient #31 has drug-resistant unclassified epilepsy and some facial features (broad nasal bridge, high arched palate): two single nucleotide variants of uncertain significance of *TBC1D24* were identified on the maternal allele (chr16:2546328G>A; chr16:2546851G>A, non-coding). Patient #32 has severe epilepsy with myoclonic atonic seizures and a heterozygous 16p13.3 duplication of 407 Kb including *TBC1D24,* identified by array-CGH. The clinical relevance of this copy number variant is uncertain, as 22 genes, including *TBC1D24*, reside in this interval. Nonetheless, it is worth noting here that overexpression of *TBC1D24* resulted in a marked increase in neurite length and arborisation in vitro,^e4,e7^ indicating a possible adverse effect of gene duplication.

The homozygous mutation p.Glu153Lys, in the two siblings with familial infantile myoclonic epilepsy without evidence of hearing impairment (#8a-b), was recently identified as part of compound heterozygosity in a Moroccan family with recessive non-syndromic hearing loss.^e8^ None of the other six *TBC1D24* mutations previously identified in families with non-syndromic hearing loss (p.Asp70Tyr; p.Ser178Leu; p.Arg214His; p.Lys266Asn; p.Arg293Pro; p.Val445Val.fs32)^e8-11^ was present in our cohort. We note that two individuals in one of the families with recessive non-syndromic hearing loss due to p.Asp70Tyr mutation have a history of seizures, though the authors suggest that this association is coincidental^e10^.

**e-References.**

e1. Brewer GJ, and Torricelli, JR. Isolation and culture of adult neurons and neurospheres. Nat. Protocols 2007;2:1490-1498.

e2. Sevgi EB, Saygi S, Ciger A. Eye closure sensitivity and epileptic syndromes: A retrospective study of 26 adult cases. Seizure. 2007;16:17-21.

e3.Campeau PM, Kasperaviciute D, Lu JT, et al. The genetic basis of DOORS syndrome: an exome-sequencing study. Lancet Neurol. 2014;13:44–58.

e4.Falace A, Filipello F, La Padula V, et al. TBC1D24, an ARF6-interacting protein, is mutated in familial infantile myoclonic epilepsy. Am J Hum Genet. 2010;87:365–370.

e5. Milh M, Falace A, Villeneuve N, et al. Novel compound heterozygous mutations in TBC1D24 cause familial malignant migrating partial seizures of infancy. Hum Mutat. 2013;34:869–872.

e6.Bilo L, Peluso S, Antenora A, et al. Parkinsonism may be part of the symptom complex of DOOR syndrome. Parkinsonism Relat Disord. 2014;20:463-465.

e7.Corbett MA, Bahlo M, Jolly L, et al. A focal epilepsy and intellectual disability syndrome is due to a mutation in TBC1D24. Am J Hum Genet. 2010;87:371–375.

e8.Bakhchane A, Charif M, Salime S, et al. Recessive TBC1D24 Mutations Are Frequent in Moroccan Non-Syndromic Hearing Loss Pedigrees. PLoS One. 2015;10:e0138072.

e9.Azaiez H, Booth KT, Bu F, et al. TBC1D24 mutation causes autosomal-dominant nonsyndromic hearing loss. Human mutation. 2014;35:819-823.

e10.Rehman AU, Santos-Cortez RL, Morell RJ, et al. Mutations in TBC1D24, a gene associated with epilepsy, also cause nonsyndromic deafness DFNB86. Am J Hum Genet. 2014;94:144-152.

e11.Zhang L, Hu L, Chai Y, Pang X, Yang T, Wu H. A dominant mutation in the stereocilia-expressing gene TBC1D24 is a probable cause for nonsyndromic hearing impairment. Human mutation. 2014;35:814-818.
