# Supplementary material for: TBC1D24 genotype–phenotype correlation: Epilepsies and other neurologic features
Source: Neurology. 2016 Jul 5;87(1):77–85. doi: 10.1212/WNL.0000000000002807 (PMC4932231; doi:10.1212/WNL.0000000000002807)
Supplement: Videos [file supp_87_1_77_v2_index.html]

TBC1D24 genotype–phenotype correlation — Videos 

# *TBC1D24* genotype–phenotype correlation

## Videos

**Neurology® data supplements are not copyedited before publication. Published editorials and translations have been copyedited.  
 © 2016 American Academy of Neurology.  
  
 Files in this Data Supplement:**

- Video 1 - .mp4 file
- Video 2 - .mp4 file
- Video 3 - .mp4 file
- Video 4 - .mp4 file
- Video 5 - .mp4 file
